# A Robust End-to-End IoT System for Supporting Workers in Mining Industries

**DOI:** 10.3390/s24113317

**Published:** 2024-05-22

**Authors:** Marios Vlachos, Lampros Pavlopoulos, Anastasios Georgakopoulos, Georgios Tsimiklis, Angelos Amditis

**Affiliations:** Institute of Communication and Computer Systems, 157 73 Athens, Greece; lampros.pavlopoulos@iccs.gr (L.P.); a.georgakopoulos@iccs.gr (A.G.); georgios.tsimiklis@iccs.gr (G.T.); a.amditis@iccs.gr (A.A.)

**Keywords:** Internet of Things, smart devices, wearables, localization, edge processing, data warehouse, sensor networks, real-time monitoring, mining industry, worker safety

## Abstract

The adoption of the Internet of Things (IoT) in the mining industry can dramatically enhance the safety of workers while simultaneously decreasing monitoring costs. By implementing an IoT solution consisting of a number of interconnected smart devices and sensors, mining industries can improve response times during emergencies and also reduce the number of accidents, resulting in an overall improvement of the social image of mines. Thus, in this paper, a robust end-to-end IoT system for supporting workers in harsh environments such as in mining industries is presented. The full IoT solution includes both edge devices worn by the workers in the field and a remote cloud IoT platform, which is responsible for storing and efficiently sharing the gathered data in accordance with regulations, ethics, and GDPR rules. Extended experiments conducted to validate the IoT components both in the laboratory and in the field proved the effectiveness of the proposed solution in monitoring the real-time status of workers in mines.

## 1. Introduction

The most crucial issue for mining industry workers is the continuous monitoring of both their health and their safety status in order to ensure their well-being and prevent undesired situations such as working accidents and occupational hazards. To achieve this goal, there are a lot of parameters that should be monitored, including environmental temperature and humidity, respiratory health by measuring the concentration of harmful gases, dust and particulate matter, noise levels, physical activity, fatigue, biometric data (heart rate, blood pressure, etc.), location of the workers, etc. However, the specific parameters that need to be monitored depend on the type of mining environment and the special conditions observed there. Thus, recent research has been focused on dealing with these issues by monitoring different sets of parameters and adopting various approaches.

To understand the context, challenges, and also constraints involved, an investigation was conducted among the various stakeholders. This included manufacturers, operators, and standards and regulation experts. Subsequently, a number of issues were identified, which could be potentially addressed using smart technology.

With these in mind, the goal was to design and implement a smart garment that could determine a worker’s location within the mine and inform a central system, represented by an IIoT platform, about the health and environmental status of the worker, notifying him by alerts of undesirable situations which require some action either from the worker or from the safety department of the mine.

The design was constrained by a number of issues, analytically described in Section 3. By fulfilling the desired requirements and dealing with the major constraints, a robust end-to-end IoT system for supporting workers in mining industries was designed, developed, and evaluated both in the laboratory and in real mining environments.

Based on the aforementioned, the main contributions of this paper are as follows:

- Demonstrates the benefits and viability of localization techniques in monitoring workers in mining environments.
- Demonstrates the ability to efficiently interact with the system for receiving alerts or warnings crucial for the safety of the workers and protect users under difficult conditions.
- Demonstrates that such a system can be built in a reliable and cost-effective way, allowing for adoption at scale.

The rest of the paper is organized as follows: Section 2 provides a comprehensive review of the existing literature, Section 3 contains a detailed description of the architecture and implementation, Section 4 presents the experimental results, Section 5 discusses outcomes, and, finally, Section 6 provides the conclusions reached.

## 2. Related Work

In [1], a dual-section system is introduced, designed for monitoring mineworkers’ status and comprehensive environmental conditions. Within the mine labor area, air pollution arises from emissions of particulate matter and gases such as sulfur dioxide (SO_2_), nitrogen dioxide (NO_2_), and carbon monoxide (CO). The system employs two smoke sensors to detect various smoke levels and semiconductor gas sensors to measure the concentration of hazardous gases. When smoke sensor readings exceed the safe threshold, the microcontroller activates an alert via a buzzer and transmits the data to the monitoring section using a LoRaWAN module, where the information is displayed on a webpage.

In [2], the authors describe the National Institute for Occupational Safety and Health’s (NIOSH) implementation of an IoT-based intelligent machine monitoring system. This system was rolled out in two phases: the initial phase involved deploying a proof-of-concept system at a concrete batch plant, while the second phase expanded the system with additional sensors, enhanced safety and performance metrics, proximity detection, and predictive failure analysis capabilities.

The work in [3] focuses on developing a real-time surveillance helmet equipped with IoT sensors. These sensors provide early warnings for hazards such as fire, silicosis dust, temperature extremes, and harmful gases, thereby reducing health risks for workers. The helmet also includes a GPS tracker to monitor a miner’s current location.

An IoT-based Dynamic Sensor Information Control System (IoT-DSICS) is proposed in [4] to address issues such as warm humidity, precipitation, and unhealthy carbon emissions in coal mines. This system integrates the Industrial Internet of Things (IIoT) with sensor networks and control systems, enhancing the safety and efficiency of coal mining operations. It uses an IIoT Wi-Fi microcontroller to manage prototypes, activate ventilation fans, and trigger alerts in the Pittsburgh Investigation of Mine, aiming to lower operational costs and improve safety.

In [5], a framework named Smart-SAGES is presented for making underground support systems smarter through IoT implementation. The paper outlines security challenges in the integration of IoT with underground mining operations, emphasizing the need for clear security goals. It suggests that blockchain technology could mitigate cyber-attacks in this context. The study identifies vulnerabilities such as information disclosure and denial-of-service attacks and recommends incorporating security measures from the outset of system development to ensure secure data transmission and storage.

In [6], a systematic review based on a comprehensive search of databases like Web of Science, Google Scholar, Scopus, and ScienceDirect, using keywords related to mining accidents, coal mining injuries, human error, and intelligent mining, is presented. The review examines 59 papers in detail, identifying safety issues in coal mining and the impact of IoT. These issues are categorized into general safety concerns, environmental factors, and mining information technology. Despite mechanization and automation improving safety, productivity, and cost in coal mines, human factors such as inadequate skills, inexperience, perceptual errors, and unsafe behaviors remain leading causes of injuries. The review suggests further research into human factors using psychological models and calls for attention to the environmental impact of abandoned mining sites.

Finally, in [7], the potential of IoT to enhance real-time platforms for remote monitoring and operation in complex production systems with minimal human intervention is discussed. This capability is particularly advantageous for hazardous industries like mining, as it can improve the safety of personnel and equipment while reducing operational costs. The study suggests that a fully automated smart mine could be realized over time by progressively leveraging IoT technologies. Currently, various sensors are employed in mining activities, including geophones for exploration and blast control, piezometers for dewatering, and toxic gas detectors for frontline safety. However, achieving a fully integrated automated system remains challenging due to limitations in communication infrastructure, data management, and storage. Additionally, the reluctance of mining companies to abandon traditional methods in favor of untested new technologies further hinders progress. The study reviews the current adaptability of the mining industry to IoT systems and examines its development trajectory. It identifies significant challenges, such as infrastructural limitations and industry resistance to change, and provides recommendations for developing a comprehensive model. This model would cater to various mining segments, including exploration, operation, and safety, by utilizing flexible technologies like wireless sensor networks and implementing global data management strategies.

Having the above in mind, and especially the outcomes of studies [6,7], in this paper, an integrated system overcoming the infrastructural limitations in communication, data management, and storage is presented. According to our knowledge, the proposed system is unique in its capability to function in extremely adverse conditions, both for open pit and underground mines. The use of IoT wearable devices, such as smart garments, proposed in this paper can simplify complex data collection, even if communication is broken. This, in conjunction with the near-real-time monitoring IIoT platform, can provide a more proactive approach that will enhance the safety of workers in mining environments while simultaneously reducing accidents and improving the overall performance and efficiency of the system. 

## 3. System Architecture

### 3.1. Device Layer

#### 3.1.1. Smart Garment System

The objective of this work is to develop an innovative smart garment that will be deployed in both open-pit and underground mines. The smart garment development ensures endurance in the harsh conditions of the physical mines, with embedded miniaturized sensing devices assessing OHSE parameters, physical and chemical agents (e.g., temperature, humidity, gases, and radiation), measuring biometrics data (e.g., heart rate, sweat, and body temperature) assessing the health condition or stress of the personnel and improved situational awareness. The smart garment that was developed is in the form of a vest/jacket comprising a number of subsystems (smart garment hub, headphones, wristband) in order to meet end users’ acceptance criteria.

The wearable solution that has been developed for mining environments achieves:

1. Minimal overall weight;
2. Long lifetime using rechargeable batteries;
3. Ruggedized design to endure operation in harsh environments.

**Figure 1:**
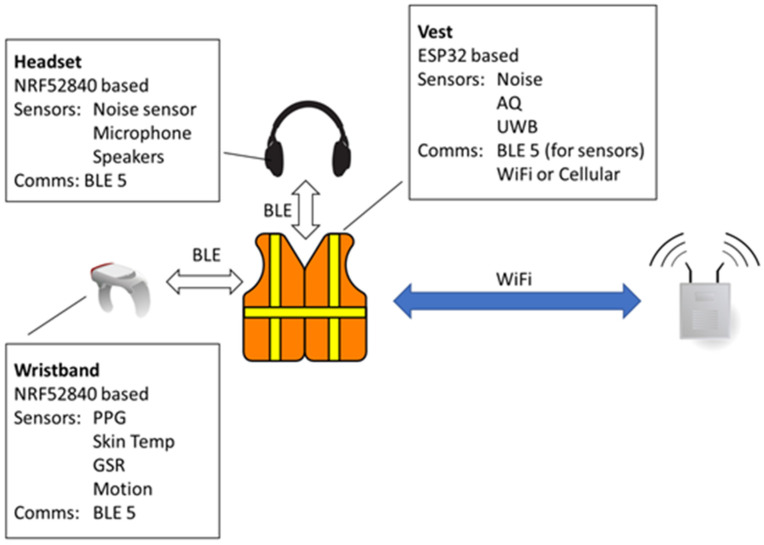
High-level smart garment architecture.

We have followed the architecture for smart garment subsystems, including components, sensors, communication devices, and computational needs, as shown in the high-level diagram of Figure 1. This can be described as a hierarchical architecture with the following components: a number of sensing devices carrying a multitude of sensors (headset, wristband, and/or AQ nodes), connected through low-power wireless BLE interfaces to a central “smart garment hub” placed on the vest. The latter is responsible for gathering the information from the devices, gathering additional information through sensors integrated into the hub itself, processing the data, and communicating with the backbone through a standard Wi-Fi interface.

This architecture is dictated by a number of factors. From the end-user perspective, the smart garment has to provide a number of different sensing functional capabilities, as summarized in the following:

- Sensing: The smart garment should gather biometric data from individuals (heart rate, heart rate variability, skin temperature, skin conductivity, and motion), environmental data (temperature, humidity, and noise levels), air quality data (CO, CO_2_, and VOC), audio communications, and voice processing/recognition.
- Processing: The smart garment should have enough processing power and memory to be able to run edge computing algorithms that reduce the total amount of data exchanged over the wireless medium. Furthermore, enough processing power is needed for the implementation of the AI algorithms of the smart earplug.
- Communication: Information from the smart garment should be available over standard communication protocols such as BLE or Wi-Fi.
- Autonomy: All smart garment components should be able to last over at least a working shift (12 h) within the mine before any recharging.
- Scalability: Although the total number of persons and equipment simultaneously located within a mine is limited to a few dozen, in future deployments it would be expected that such systems could scale up significantly, creating a complete digital footprint of all smart garment users, and thus offering a complete view of person-operations within the mine.
- User Acceptance: The smart garment devices should meet strict weight and size requirements in order to be usable and acceptable in harsh working environments, such as the ones met in both open-pit and underground mines.

The aforementioned requirements are quite challenging and oftentimes contradictory. For example, if we increased the autonomy of sensing devices by using high-capacity batteries, this would increase the weight and size of the design and consequently affect user acceptance in a negative way. If we decreased the edge computing capabilities of the devices by running limited (if any) pre-processing algorithms on the gateways of the network, this would increase the communication burden on the wireless network and reduce the autonomy of the devices.

We have therefore concluded that the most efficient architecture for meeting all the requirements outlined by the end users is the proposed architecture described in Figure 1, for the following reasons:

- Sensing scalability and network complexity: Each sensing device is restrained from sending raw data samples directly to a network gateway. This reduces the complexity of the network and ensures that all communications are controlled and scheduled by the smart garment hub, reducing power consumption even in the case when there is no connectivity with the backbone network. This modular design is more versatile since sensors on the smart garment can be changed (e.g., based on performance), added (to improve accuracy and/or efficiency), or removed/replaced in a more straightforward fashion.
- Autonomy and user acceptance: We retain the small batteries on the headset and wristband where the weight and size requirements are stricter, and we can have larger batteries on the vest, where data gathering and processing is conducted. Moreover, since the BLE links between the edge devices (headset, wristband) have a very short range, we can set the BLE class to 0, minimizing the transmission power, thus minimizing their power consumption.
- Processing and communication trade-off optimization: We are given the flexibility to optimize the amount of processing effort performed on the edge devices (headset, wristband) and on the smart garment hub, thus controlling the total amount of information that has to be transmitted over the wireless network.
- Communication flexibility: The chosen architecture provides great flexibility as the smart garment can adapt to new use cases or to different communication infrastructures. For instance, if the backbone infrastructure inside the underground mines is replaced by a wireless system other than Wi-Fi, or if the smart garment needs to be used in outdoor environments (e.g., in open pits), where cellular connectivity is more suitable, then the proposed architecture can adapt by simply changing the smart garment hub communication module, without having to change the communication links of the other subsystems.

The architecture described in the previous paragraph can be broken down into a set of core subsystems, which will be described in detail in the following sections:

- Vest: The smart garment hub, which collects the data from the edge devices through BLE interfaces, encompassing sufficient processing power, memory, and battery capacity to facilitate edge computing algorithms and analytics tasks, and is responsible for all communications with the backbone network.
- Wristband: This smart garment component is responsible for all biometric data gathering to monitor several health-related parameters.
- Headset: The headset is responsible for all audio-related functionalities, including noise level measurements, audio communications, and voice processing/recognition.

##### Smart Garment Vest/Hub

The hub subsystem is the “heart” of the smart garment system. At its core lies an ESP32 System-on-Chip (SoC) [8], which is responsible for gathering information from the smart garment sensors either through BLE connections (from the aforementioned subsystems) or through its own analog and digital interfaces (from sensors placed locally on the smart garment hub). It is also responsible for processing and running local AI/ML algorithms and for communicating with the backbone network over a Wi-Fi interface.

**Figure 2:**
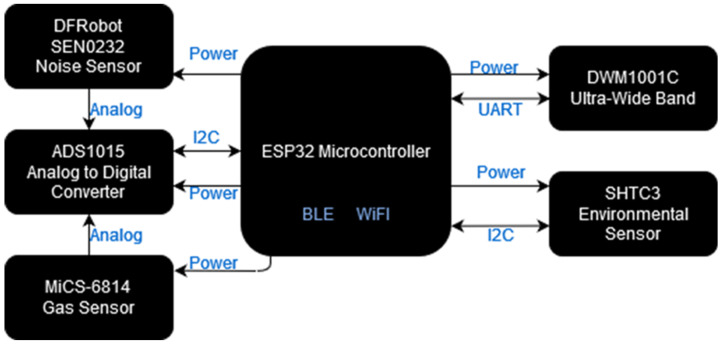
Architecture of the smart garment hub.

**Figure 3:**
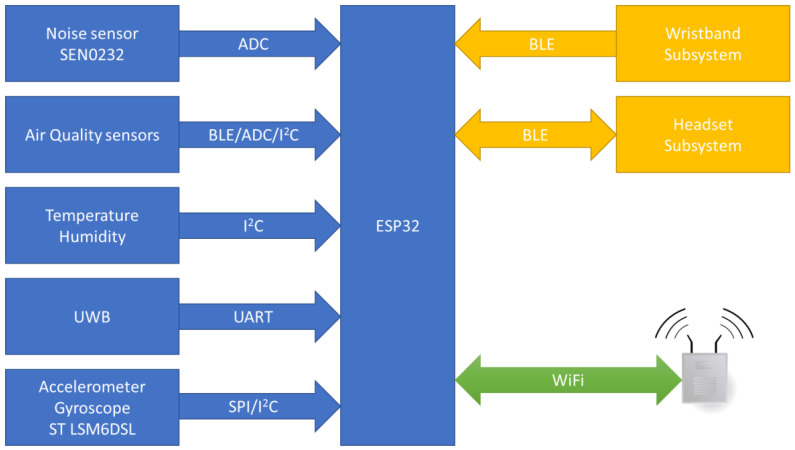
High-level architecture of the smart garment hub.

The smart garment hub consists of the following components: the ultra-wideband (UWB) component [9] that provides indoor localization; the sensors component, which includes the noise level sensor DFRobot SEN0232, the air quality sensor MiCS-6814 (CO, NH_3_, NO_2_), and the environmental sensor SHTC3 (temperature, humidity); and the main microcontroller unit (MCU) that provides the local processing unit and interfaces with peripheral systems. The latter is also responsible for communicating with the backbone through a Wi-Fi interface. After conducting in-lab measurements of the total power consumption of the smart garment hub, a 2500 mAh lithium polymer rechargeable battery was chosen for the power supply in order to achieve the desired autonomy of a 12-h working shift. The architecture of the smart garment hub is illustrated in Figure 2, while Figure 3 summarizes the different interfaces used for communicating with the various components.

The smart garment hub also works as the wristband’s gateway, as it is the responsible subsystem for acquiring all the gathered biometric data from it via the BLE protocol. After the acquisition of all the time series of these measurements is completed, it formats them appropriately into JSON [10] files and transmits them to the IIoT platform via the MQTT protocol [11]. The smart garment hub is not a hardware component of the smart wristband and subsequently is not integrated into the wristband’s board. Nevertheless, it is necessary for the successful accomplishment of the wristband’s purpose, which is the reliable real-time monitoring of coal workers’ health state.

##### Smart Wristband

**Figure 4:**
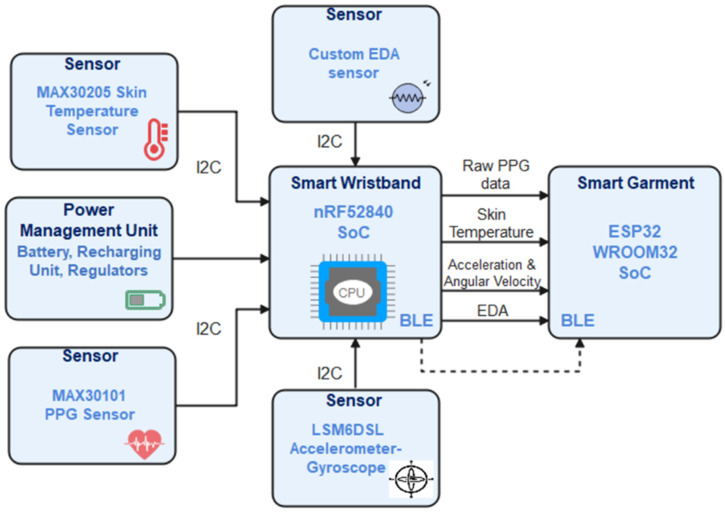
Wristband’s architecture.

The assessment of mine workers’ health state by monitoring essential vital signs, such as heart rate, oxygen saturation level, sweat, and body temperature, is an essential and required functionality of the smart garment. To achieve that, the development of a miniaturized, reliable, and long-life smart wristband has been completed. The architecture of the wristband is presented in Figure 4.

The architecture of the wristband subsystem is composed of the following main components:

- Main Processing Unit: The selection of a microcontroller capable of hosting the wristband’s firmware, providing Bluetooth Low Energy communication, multiple digital communication interfaces, and relatively low power consumption was a priority. Furthermore, the main processing unit should provide enough computational and memory resources to enable the signal processing functionality and the storage of time series of data. With all the above in mind, the nRF52840 SoC of Nordic Semiconductors [12] has been chosen as the main processing unit of the device. The nRF52840 accommodates a powerful ARM ^®^ Cortex ^®^ -M4 32-bit processor with FPU at 64 MHz along with 1 MB of Flash and 256 KB RAM and supports BLE 5 protocol that makes it sufficient for the proposed development of the wristband.
- PPG biometric sensor: The most common non-obtrusive way to monitor the heart’s function on the wrist is an optical sensor that uses PPG technology. PPG is a low-cost and non-invasive optical technique used to detect volumetric changes in the blood. These volumetric changes can be used to detect the systolic peaks and calculate vital signs, such as heart rate and oxygen saturation level (SpO_2_). Keeping all the above in mind, the MAX30101 [13] heart rate and pulse oximeter sensor from Maxim Integrated, which provides a single packaged module including all required optical elements, has been selected. These elements are three integrated LEDs and two photodetectors, plus noise filters, ambient light rejection, and analog-to-digital converters for I^2^C communication with the MCU.
- Skin Temperature sensor: Besides the basic vital signs provided by the PPG sensor, skin temperature is another important value that can assist in the assessment of a miner’s health state by identifying sudden increases and decreases in skin temperature. The reliability of such measurements and the low power consumption of the module were the main criteria for choosing this sensor. We concluded that the best solution was the MAX30205 [14] from Maxim Integrated, which provides an accuracy of 0.1 °C, low power consumption, and I^2^C interface communication.
- Accelerometer and Gyroscope (IMU 6): The movement of the wrist has been proven to be a usual source of noise for data acquired from an optical PPG-based sensor [15], which subsequently affects the extracted vital signs. Using the accelerometer and gyroscope, the acceleration and angular velocity of the wrist on each of the three axes can be measured and define the reliability of the biometric data. In addition to that, the values acquired by the IMU can be used to extract useful conclusions regarding the intensity of the miner’s work. The LSM6DL from ST Microelectronics [16], which is a very low-power IMU 6 SoC, has been integrated into the wristband to provide the aforementioned measurements with high accuracy. The IMU interacts with the MCU via I^2^C protocol.
- EDA sensor: The electrodermal activity, also known as galvanic skin response (GSR), describes the conductance of the human skin and its variations that can be caused by sweating and other stimuli closely related to human fatigue. For this reason, EDA is a valuable vital sign for the assessment of the human health state, especially in hard working conditions like the ones sometimes existing in the mines. To monitor the EDA on the workers’ wrists, an electronic circuit that applies a harmless and stable electric current via electrodes attached to the wristband has been implemented. The same circuit measures the voltage difference on the two electrodes and then amplifies it using two operational amplifiers (OpAmps). Finally, an ADC SoC converts the analog signal to digital and transmits it to the MCU via I^2^C.
- Power Management Unit: The wristband should be able to operate continuously for the whole period of a miner’s shift (8–10 h) and be re-used every day for many months. To achieve that, a power management unit that consists of a stable charging and overvoltage protection circuit along with a lithium polymer recharging battery is integrated. Using this capability, the end users (miners) can recharge the wristband after the completion of every shift by simply plugging in a charger via a USB cable. Furthermore, a buck–boost DC/DC voltage converter that outputs 3.3 V and a step-up DC/DC converter that outputs 5 V have been used to supply appropriately the MCU and sensors. Another important part of the power is the magnetic reed switch. The magnetic reed switch works as a power switch for the smart wristband and connects or disconnects the battery with the rest of the circuit.

Each vital sign provided by the biometric sensors described above (heart rate, SpO_2_, EDA, skin temperature) has been calibrated and tested via validation tests that are described in the Section 4.

##### Smart Earplugs

**Figure 5:**
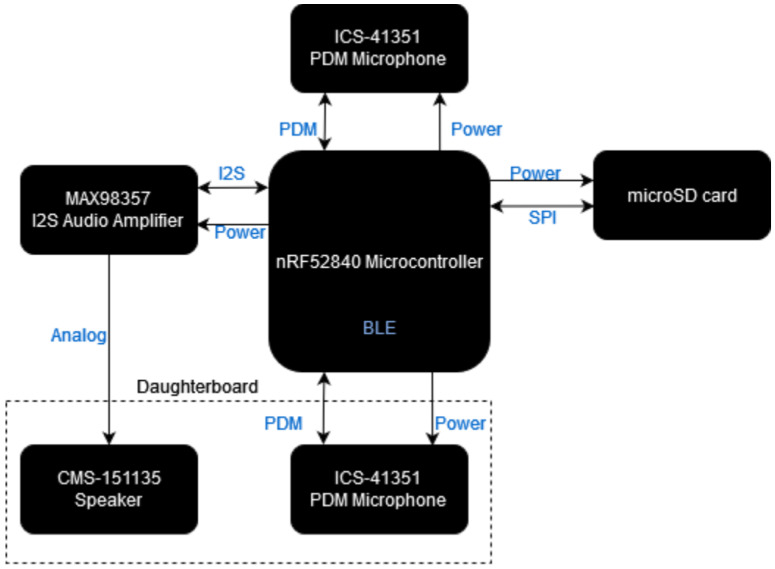
Architecture of the smart earplugs.

The smart earplugs are responsible for measuring the exposure of the mine workers to excessive noise levels while also providing an audio chatbot. Their high-level architecture is presented in Figure 5. It consists of (a) a main module mounted on the outer side of the protective earmuffs, containing the microcontroller unit, which is responsible for communicating with the smart garment hub; a microSD card, which holds the audio files; an audio amplifier for the speaker; a microphone for capturing the keywords, while also measuring the noise level outside of the protective earmuffs; a lithium polymer for power supply; and (b) a daughterboard module mounted on the inner side of the protective earmuffs, containing a microphone for measuring the noise level which passes from the protective earmuffs; and a speaker for audio feedback to the user.

#### 3.1.2. Edge Processing Functions

Various functions are running on the edge devices in order to achieve the goal of reducing the amount of data transferred over the wireless (BLE or Wi-Fi) interfaces. The functions, the architecture, the hardware on which software runs, and the wireless network that is used are described in the section describing the corresponding device. Table 1 summarizes the edge processing functions categorized by the device in which they are running.

#### 3.1.3. Communication and OTA Programming

##### Communication

As already mentioned, the communication between the smart wristband and the smart vest is established via BLE. This protocol shares many similarities with the classic Bluetooth protocol, such as being covered by the Bluetooth specification [34] and operating within the 2.4 GHz ISM band. Nevertheless, they are two distinct, incompatible protocols. The BLE protocol is designed for applications that do not require handling a lot of data but require an extended battery life. In order to use the protocol in the application, the Generic Attribute Profile (GATT) should be determined, defining the format of the data exposed by a BLE device as well as the procedures needed to access the data exposed by a device.

In our implementation, the wristband acts as a server BLE device, and the vest as a client device. The service that hosts the wristband has custom UUIDs and characteristics. The UUIDs used are Raw PPG, Heart Rate, SPO_2_, Accelerometer–Gyroscope, and Skin Temperature.

In order to avoid data loss during data transmission and to achieve synchronization between the vest and the wristband during the sleep and data collection mode, a sequence of commands and a relevant service were created. The sequence starts with a message from the vest in order to start measuring. When the wristband completes the measurements, it sends a notification message that it is ready to send data. The other side confirms with another message, so the data transmission starts. When the wristband stops sending a specific measurement, the vest sends back a message that informs it that all data have been sent. After this, the wristband repeats the same process for the rest of the data.

In order to send the data from the vest to the data warehouse, a connection using Wi-Fi protocol was established. The ESP32 MCU has an integrated Wi-Fi that works at 2.4 GHz frequency. Using several on-site access point devices, the vest can connect to the Internet. Several tests were performed to check the connection with the Internet and the correct data transmission with the MQTT protocol.

##### Over the Air (OTA) Reprogramming

The firmware update over the air cannot be performed using the MQTT protocol that the device was configured to use to send data, as it needs to retrieve a file, and an MQTT message broker does not support that. So, the device also needs to support the HTTP protocol to make it possible to download the binary files using an HTTP request. Thus, the device was configured to support both protocols. Regarding the OTA upgrade, the vest device checks if a new version of the firmware is available, downloads it in a separate partition of the flash, and updates the firmware.

#### 3.1.4. PCB and Enclosure Design

##### PCB Design

Printed circuit board (PCB) assemblies are a vital part of embedded systems, which vary in their function, size, and complexity so dramatically that careful planning and design are required to meet the exact requirements. Best practices for the functionality, manufacturability, and reliability of the PCBs have been adopted. The most important aspects in PCB design taken into account for smart garment systems are the needs of PCB assembly, interfaces to be supported, size, modularity and flexibility of integration, power consumption, processing capabilities, security, reliability, and cost.

**Figure 6:**
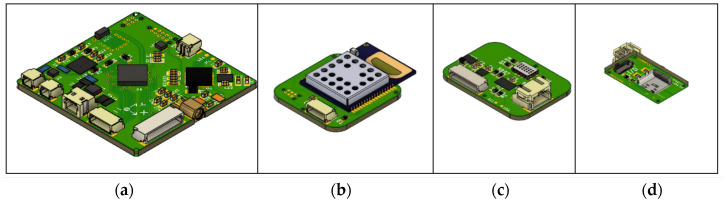
PCB 3D layouts: (**a**) Microcontroller unit, (**b**) Ultra-wideband module, (**c**) Sensor module, (**d**) Battery charger and switch.

**Figure A1:**
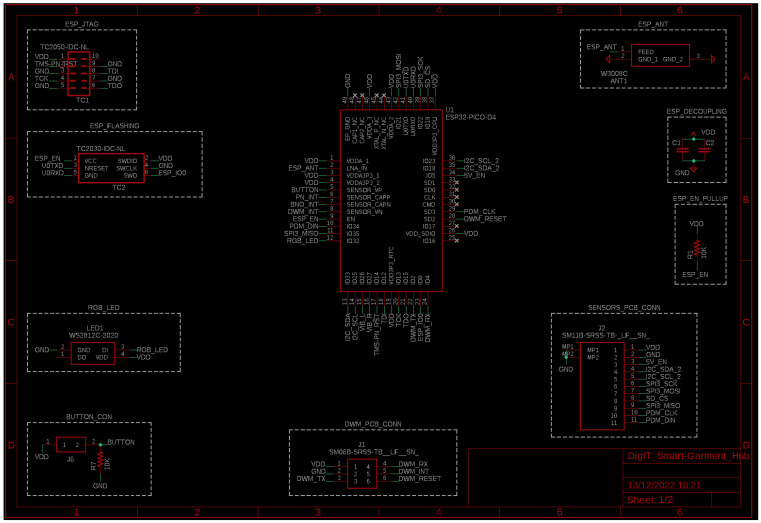
Schematic of the smart garment hub (1/2).

**Figure A2:**
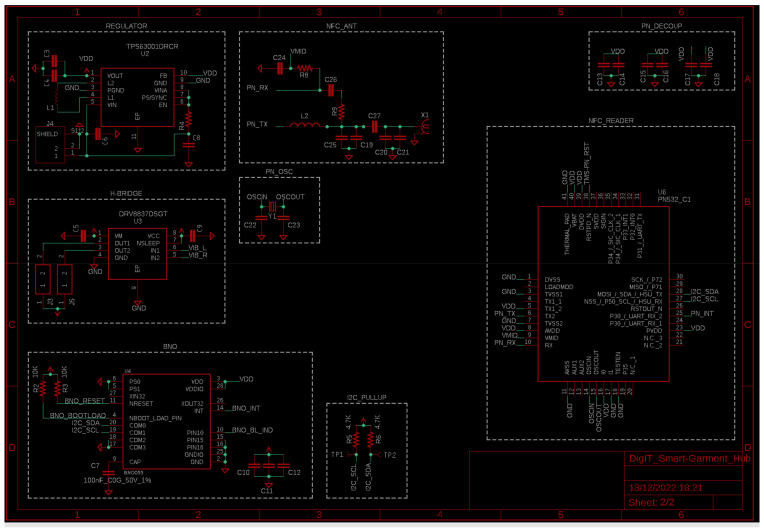
Schematic of the smart garment hub (2/2).

**Figure A3:**
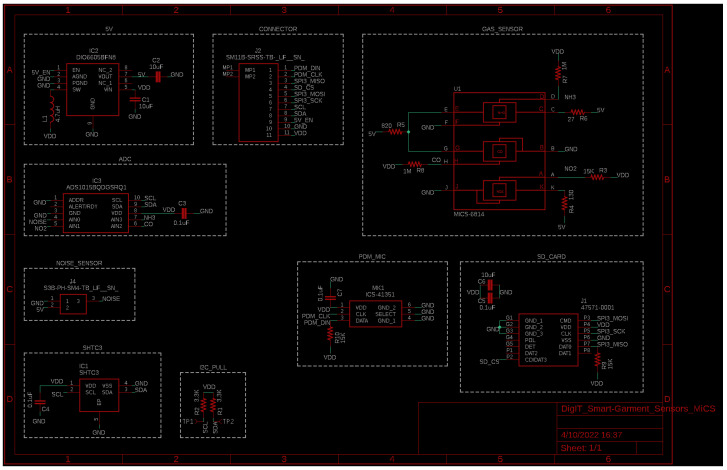
Schematic of the smart garment sensors board.

**Figure A4:**
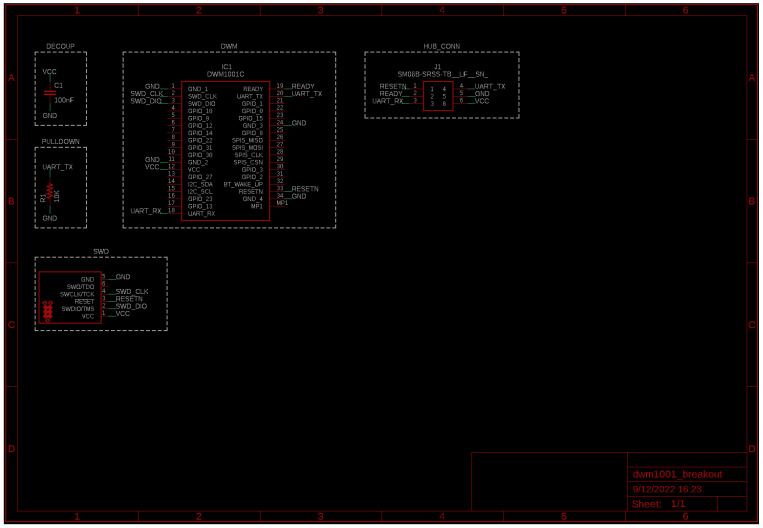
Schematic of the ultra-wideband board.

**Figure A5:**
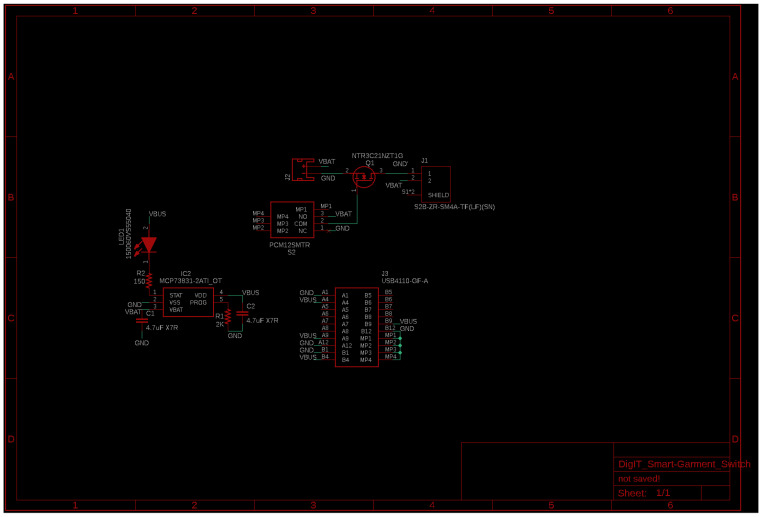
Schematic of the smart garment hub switch and battery charger.

Once all design aspects have been addressed and defined, detailed schematic drawings (Appendix A, Figure A1, Figure A2, Figure A3, Figure A4 and Figure A5) and 3D layouts (Figure 6) were created. After the design stage, the fabrication of the PCBs and the assembly of the embedded systems was conducted, while in parallel, the firmware development was implemented. Finally, functionality and reliability testing were conducted in all implementation steps. In order to produce a proof of concept and to develop the firmware while the PCBs were in the design phase, hardware development kits and evaluation boards were acquired for the components, which were selected in the analysis phase. For the design of the PCBs, EAGLE EDA (version 9.6.0) software [35] was used. For the 3D printing of the mockups, an Original Prusa i3 KM3S1 3D printer was used.

Extensive market research was conducted for each component of the smart wristband, including the MCU and biometric sensors. Development kits of proposed components were purchased to assess their efficiency and reliability through firmware development and testing. The initial prototype, though functional, faced challenges regarding size and shape, necessitating the development of a miniaturized, compact, and cost-effective PCB. The PCB design brought several benefits, including miniaturization to ensure the device’s convenience and non-obtrusiveness for mine workers, reduced costs through the use of standalone SoCs and cost-effective components, scalability for mass production, and increased reliability compared to manually assembled circuits.

**Figure 7:**
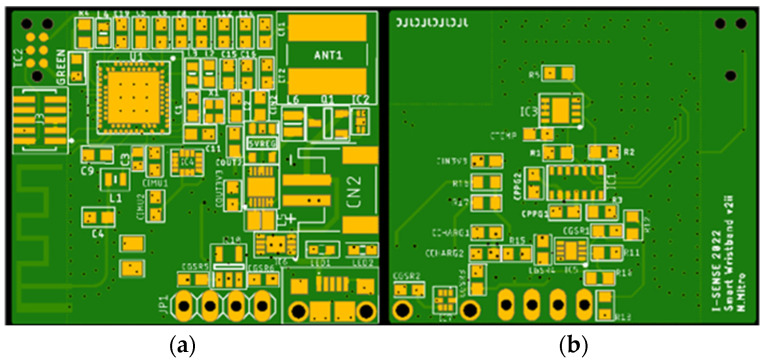
Wristband’s PCB design: (**a**) Top side, (**b**) Bottom side.

**Figure A6:**
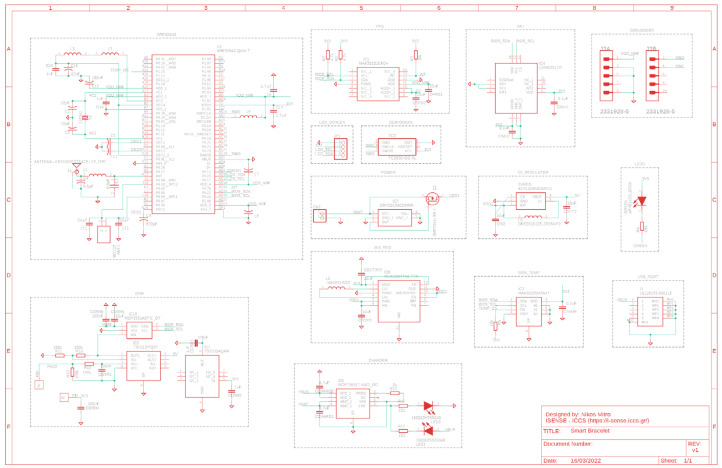
Wristband’s PCB schematic.

Similarly, the design of the smart wristband’s schematic and PCB was implemented using the Autodesk Eagle software, version 9.6.0. The electronic schematic is depicted in Appendix A (Figure A6: Wristband’s PCB schematic) and the PCB in Figure 7. The majority of the components are placed on the top side, including the MCU and its peripherals, along with the power management SoCs and the IMU 6 sensor. On the other hand, the bottom side accommodates the PPG and skin temperature sensors because both of them should be in touch with the miner’s wrist to successfully acquire reliable measurements.

**Figure 8:**
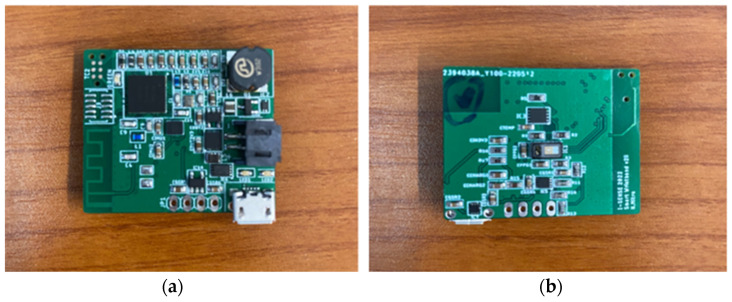
Wristband’s PCB after assembly: (**a**) Top side, (**b**) Bottom side.

Additionally, the inclusion of a magnetic reed switch served as a power switch for the smart wristband, allowing for convenient powering on and off. A green LED indicator was incorporated to signify the device’s power state. Surface mount technology (SMT) components were employed, with a total of six PCB boards successfully constructed, assembled, and tested for long-term use in mining environments. These boards are purposed to be used in mines by mine workers for long periods to gather biometric data. In Figure 8, the PCB board after the assembly and the placement of all the components are presented.

**Figure 9:**
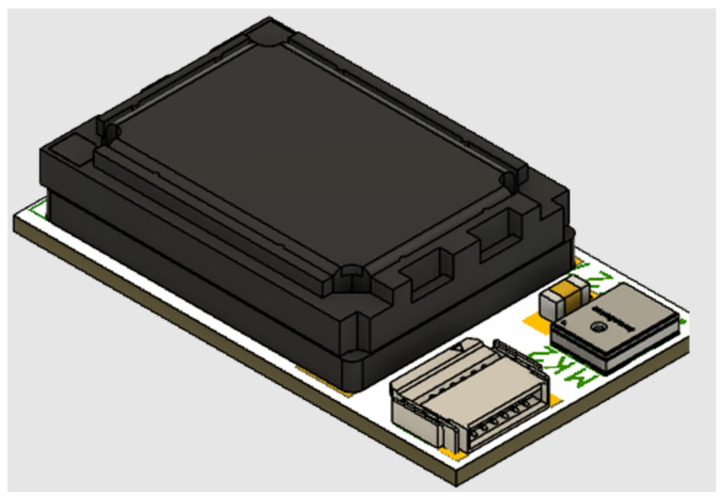
3D representation of the smart earplugs’ hardware.

**Figure A7:**
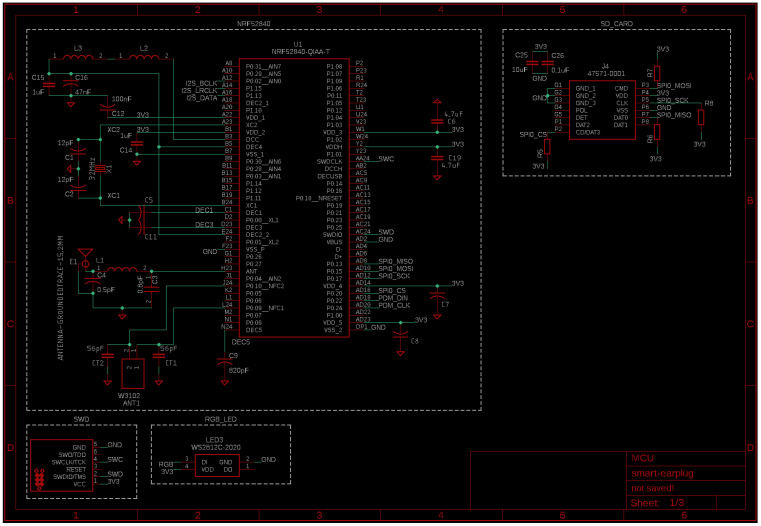
Schematic of the smart earplugs (1/3).

**Figure A8:**
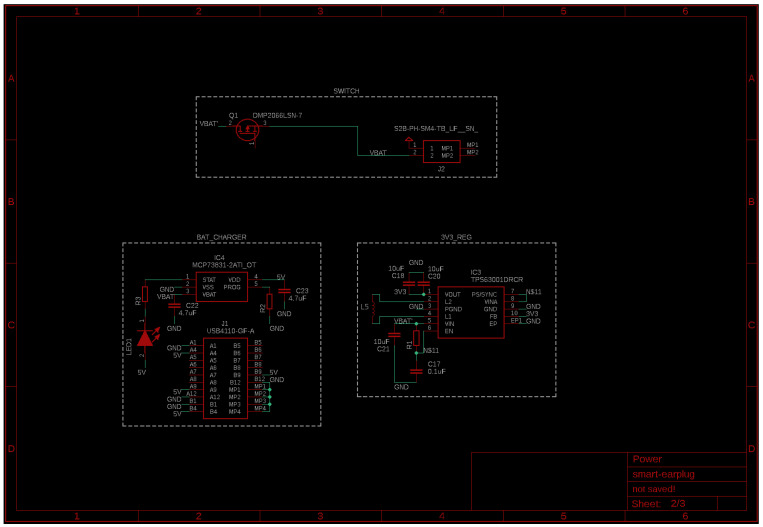
Schematic of the smart earplugs (2/3).

**Figure A9:**
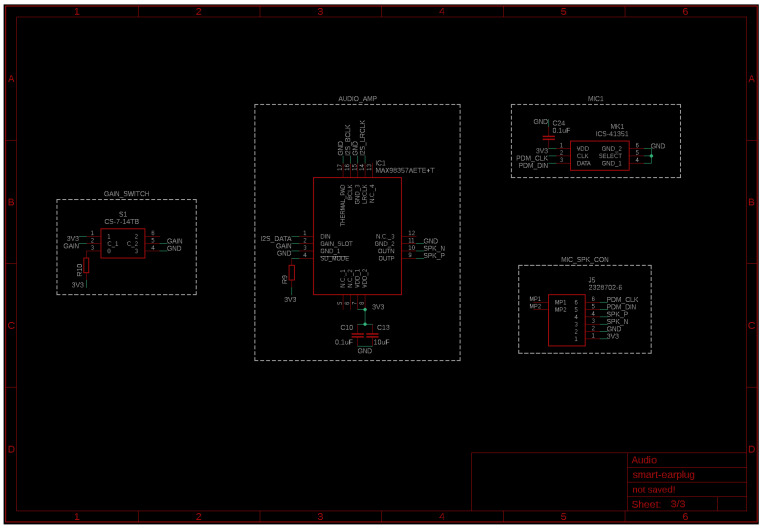
Schematic of the smart earplugs (3/3).

**Figure A10:**
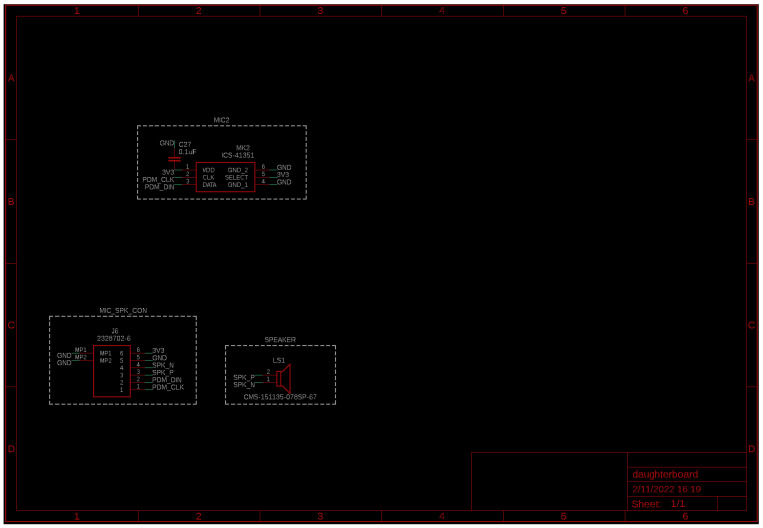
Schematic of the smart earplugs’ daughterboard.

The design of the smart earplugs schematic and PCB was implemented using the Autodesk Eagle software. The electronic schematic is depicted in Appendix A (Figure A7, Figure A8, Figure A9 and Figure A10), while the 3D representation of the PCB layout is shown in Figure 9.

##### Enclosures Design

**Figure 10:**
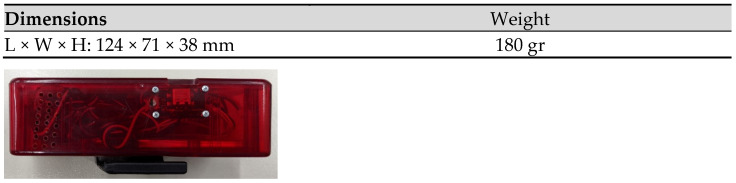
Side view with the hidden activation switch illustrated (on the left of the charging port).

**Figure 11:**
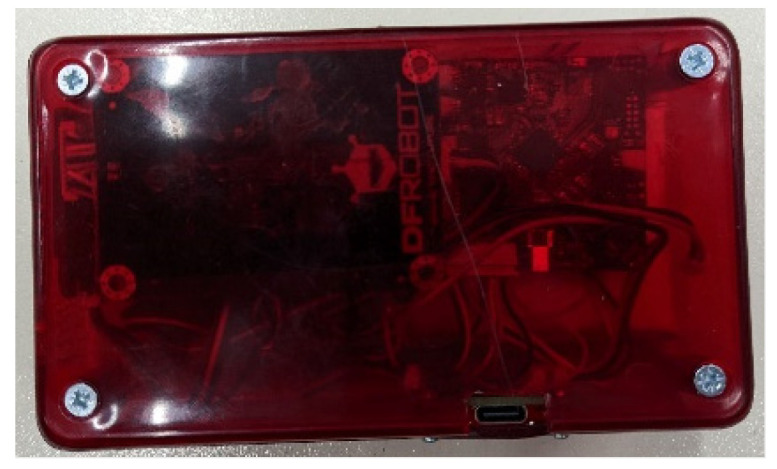
Top side of the enclosure, with the charging port (USB Type-C connector) illustrated.

**Figure 12:**
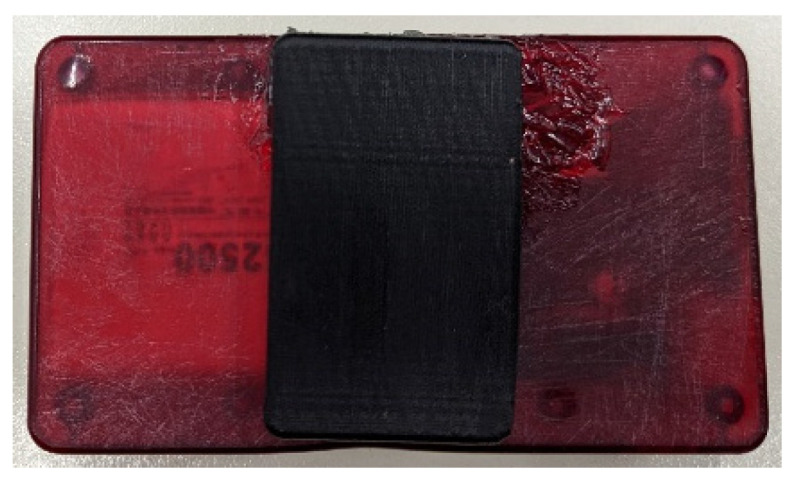
Bottom side of the enclosure with the belt clip illustrated.

For the casing of the smart garment hub, an off-the-shelf semitransparent ABS box was chosen. For the mounting of the hub, initially, a Velcro strap was used on the safety vest. After receiving feedback from mine workers, it was decided that it is more convenient to mount the hub on the belt. A belt clip was designed and 3D printed (illustrated in Figure 10, Figure 11 and Figure 12).

**Figure 13:**
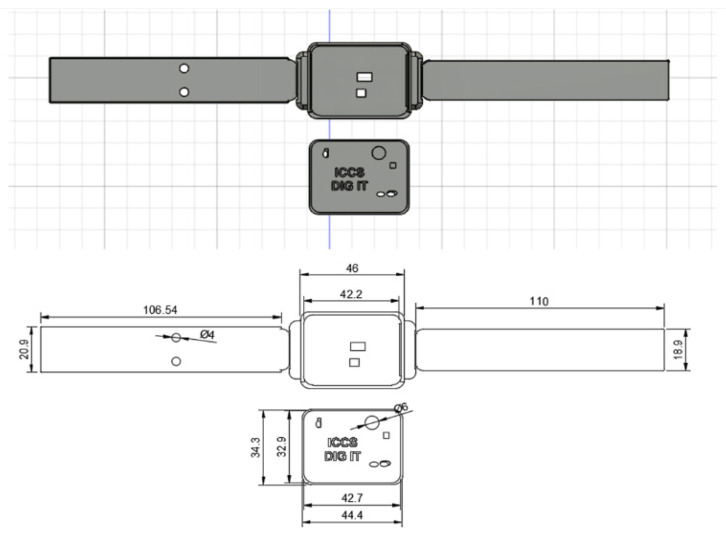
Mechanical drawing of the wristband’s casing.

A custom-made enclosure for the wristband was designed to accommodate the PCB board and the battery and has the appropriate openings for the integrated LEDs on the top, the PPG and skin temperature sensor on the bottom, and the USB port on the side. Furthermore, the fact that the two electrodes measuring the EDA should be in touch with the bottom side of the wrist made the custom design of the straps necessary. Respecting all the above requirements, the casing was designed using the Autodesk Fusion 360 (version 2.0.16490) software, and its mechanical drawing is presented in Figure 13.

**Figure 14:**
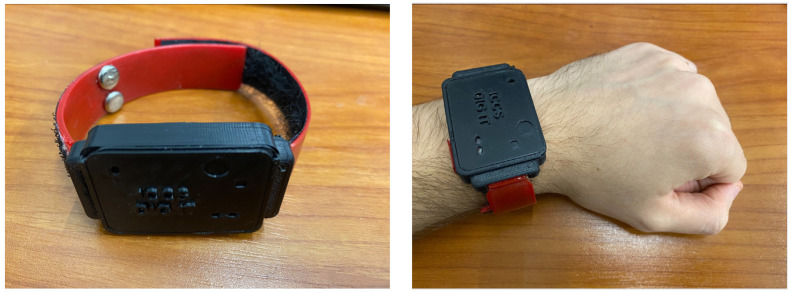
Final version of the wristband.

The Prusa i3 Mk3S 3D was used for the production of the casing. For the enclosure, acrylonitrile styrene acrylate (ASA) filament was used due to its durability and water, chemical, and strong UV resistance, which makes it suitable for indoor and outdoor environments. Regarding the straps of the wristband, the Thermoplastic Polyurethane (TPU) filament was chosen as 3D printing material as it offers not only the flexibility to be better fitted on the wrist but also sufficient compactness. As mentioned in the EDA sensor’s description, two electrodes should be in touch with the bottom side of the wrist to measure the conductance of the skin. To achieve that, the two electrodes (Ag/AgCL) were integrated on the right strap of the bracelet along with two wires required for their connection to the PCB. The addition of the electric wires inside the strap was completed after interrupting the 3D printing process and sticking them into specifically designed channels to the strap’s interior. Another feature of the wristband’s design worth mentioning is the use of adhesive Velcro tape on the straps to achieve a tight and easy connection between them for wearing. The wristband is a lightweight wearable device of 22 g. The final version of the developed wristband is depicted in Figure 14.

The developed wristband can be easily adjusted on wrists due to the adhesive Velcro tapes. Specifically, it is able to be worn on wrists with a circumference of 4 cm to 7 cm, thus accommodating the average wrist size of both males and females aged between 20 and 80+ years old (https://criticalbody.com/average-wrist-size/, accessed on 20 January 2023).

**Figure 15:**
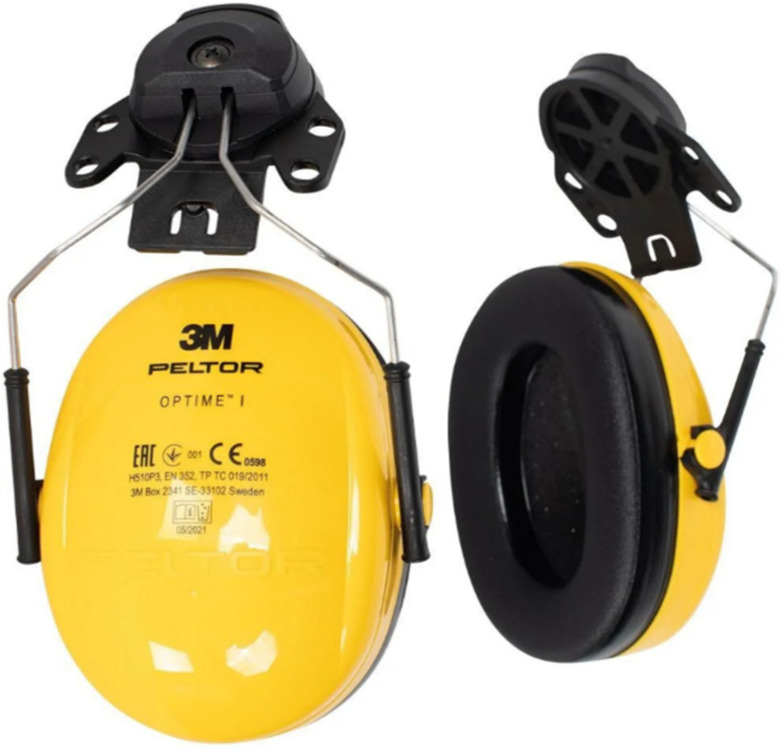
3M Peltor Optime I protective earmuffs.

To accommodate size restrictions within the earmuffs and comply with safety regulations regarding noise exposure, a modular approach was adopted for the smart earplugs. The main module is housed in an enclosure mounted on the outer side of the earmuffs (Figure 15), while the daughterboard module is mounted on the inner side. Interconnection between the two modules is facilitated by a flexible flat cable, chosen for its minimal obtrusiveness to the user during wear.

### 3.2. Platform Layer

#### 3.2.1. IIoT Platform Architecture

After examining all the system requirements, the architecture platform for the different components (sensors, devices, monitoring units, controllers, data gathering technologies, networks, etc.) is accomplished by transforming them into technical specifications. The platform architecture features real-time data collection from the distributed sensors across the worksite of the mines. Consequently, near-real-time data transmission was implemented with publish–subscribe messaging protocol technologies such as MQTT and Kafka. All the software and hardware infrastructure was designed in such a way that it will expedite and ease the work of end users. The whole architectural design is scalable, flexible, and it ensures interoperability and easy information flow between the various components and different data concepts (e.g., real-time measurements, input or output of models, economical information, etc.).

**Figure 16:**
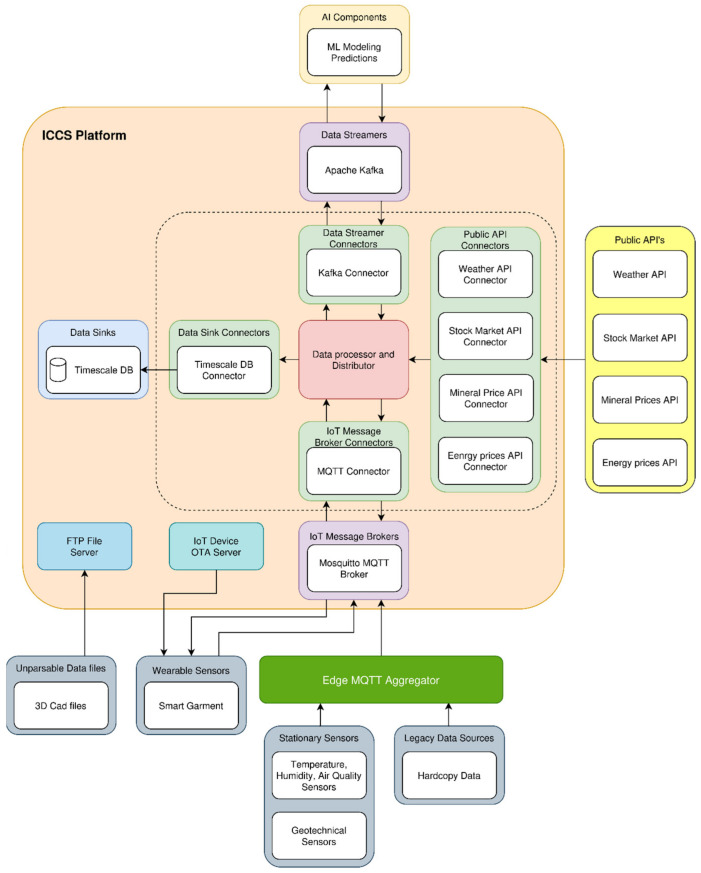
IIoT platform architecture.

Therefore, the IIoT platform stores data from multiple data sources and sends them to multiple data destinations. All of IIoT data are stored in a central location and can be accessed from all components and users. Figure 16 summarizes the high-level architecture of the IIoT platform and its basic building blocks. As shown, the IIoT platform consists of the following components: data processor and distributor, MQTT broker [36], Timescale DB [37], Apache Kafka [38], various data connectors (data streamers, data sinks, IoT message broker connectors, public API connectors), OTA server for garment updates, FTP server.

As referred before, Figure 16 depicts the high-level architecture of the IIoT platform, which consists of various building blocks. The central software component of the platform is the data processor and distributor block, which performs data processing, harmonization, and exporting to various destinations. A lot of data come to the platform and from various sources. There are data (mainly sensor data) coming from the aggregator, some from the smart garment hub, some from ML modeling input/output, and others from external sources via APIs.

Various data connectors are implemented so that data are successfully read and written by the data processor and aggregator and the various data sources and data destinations. We have tried to decouple the data transformation and routing from the connector code for many technical reasons, but mainly to enable easy scaling of the IIoT platform in the future and support a vast variety of extra data sources and data sinks. Currently, Timescale DB, MQTT, and Kafka connectors are used. Different connectors are used depending on the source of the data. Thus, the data concentrated in the smart garment are sent to the platform via the MQTT connector. The same happens also for the data gathered by the aggregator. Other data come from APIs and others by parsing files. In addition, different connectors are used for exporting the data to various destinations. Some data are available by directly querying the timescale database, while others are published in a Kafka broker to be available for consumption.

#### 3.2.2. Functional Architecture

The data processor and distributor is the central software component of the IIoT platform. All data from various data sources arrive at the data processor and distributor and are directed to various data terminals. The necessary data transformations are performed in this component depending on the data destination. The data flows are currently supported from the MQTT broker to Kafka and Timescale DB and from Kafka to MQTT broker and Timescale DB. 

Data that arrive at MQTT are from various data sources on the edge, mainly data from sensors, needing to be cleansed and harmonized so that they can be stored in various data sinks afterward. This data processing is performed in this component. Data validation is initially needed to ensure that any data packet coming from edge sensors or other devices has the expected schema. Afterward, data are transformed so that they can be stored in Timescale DB and sent to the Kafka broker. Data that arrive at the Kafka broker are mainly data coming from other cloud services, such as machine learning algorithms. Again, data validation is performed as a first step, and then data are transformed and sent to Timescale DB and the MQTT broker, especially if a data message must be sent from the cloud service to the edge device.

A further improvement will be to use a standard API in the future that can be used for the purposes of sharing sensors’ data, which is the SensorThings API [39]. SensorThings API provides a standardized and interoperable persistence layer for data gathered in real time through online sensors, data gathered offline via sensors, or measurements by lab validation.

**Figure 17:**
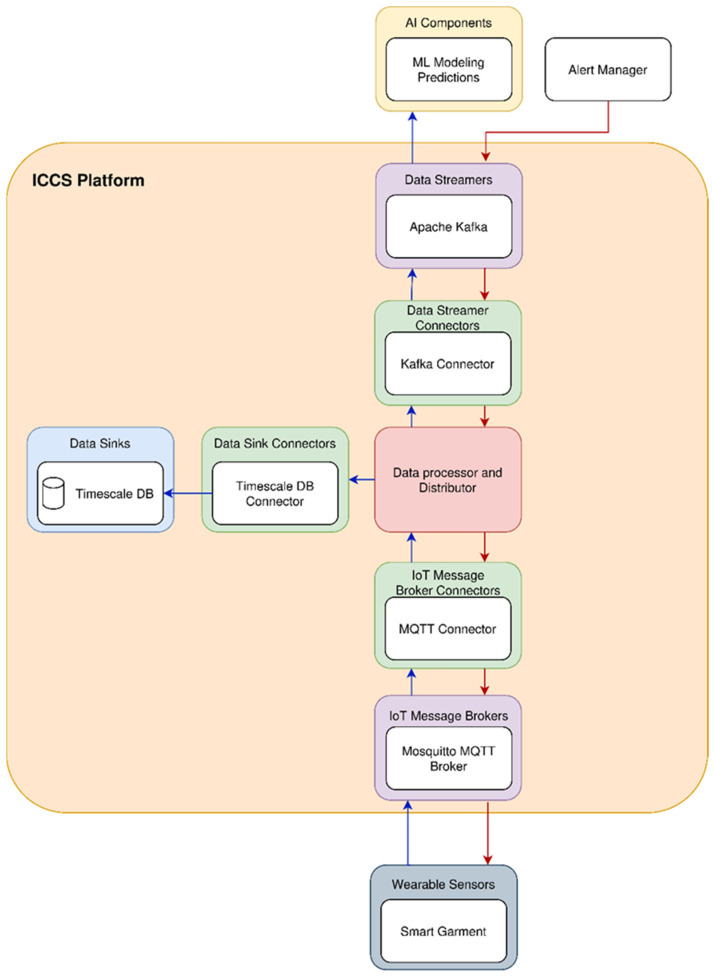
Illustration of data flow.

The following example illustrates a sample data flow from the edge to a cloud service and from a cloud service back to an edge device. As depicted in Figure 17, starting from the wearable smart garment, data relative to the human operator’s health and local environment around the operator’s location are sent to the MQTT broker from there to the data processor and distributor and then to the data sinks and data streamers, mainly Timescale DB and Kafka. From Kafka, data are transferred to an AI cloud service where insights, predictions, etc., are calculated. Then, if for any reason an alert has to be sent to the human operator from the cloud, then the alert manager that generates the alert sends the alert data packet to Apache Kafka, and from there, the data processor and distributor is configured to send the alert data packet to the MQTT broker and from there the wearable smart garment. Lastly, the alert data packet is processed by the smart garment device and plays the corresponding audio file to the speaker of the smart earplugs so that the human operator gets informed about the actions that should be performed depending on the alert type. In Figure 17, the blue lines depict the data flow from the smart garment to various data destinations, and the red lines from the alert manager to the smart garment, with all corresponding data transformations and routes configured by the data processor and distributor.

## 4. Results

Once the smart garment devices were developed, integrated, and tested, a measurement campaign for field experiments both in underground quarries and in open pits was organized. In order for the experiments to be conducted successfully, two important issues had to be resolved: GDPR/ethics and end-user training. The former was handled by seeking volunteers willing to wear such devices, participate in experiments, and fill out a consequent form relevant to GDPR regulations. To resolve the latter, a smart garment short manual was created, together with informative videos, and shared with the end users. In addition, informative meetings were conducted with the mine workers both to find candidate volunteers and to inform them regarding GDPR, ethics, and training issues.

**Figure 18:**
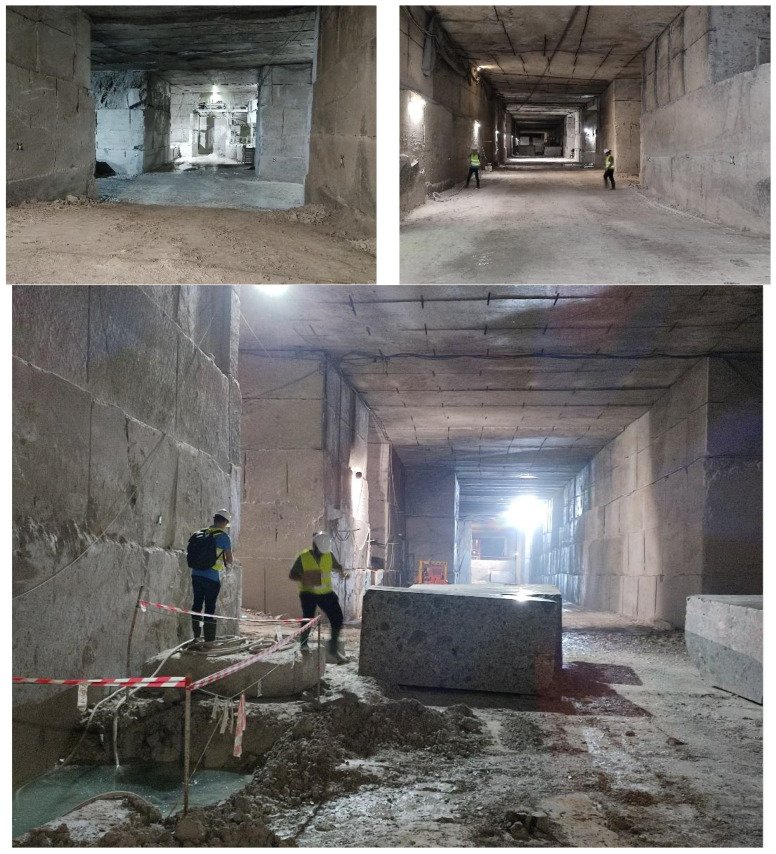
Marini-Marmi testing campaign.

**Figure 19:**
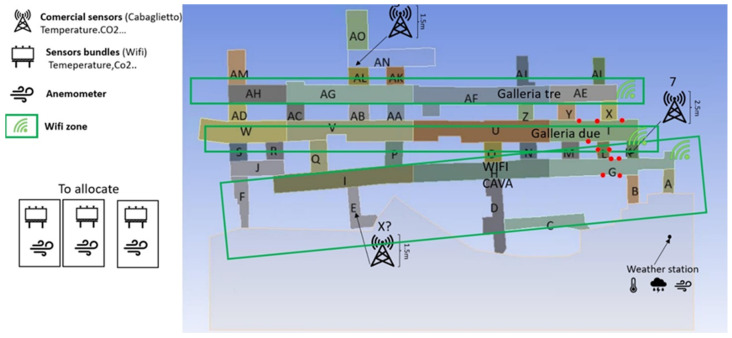
Marini-Marmi testing setup.

Underground tests were conducted in the Marini-Marmi and Kemi mines, while open pit tests were conducted in the Titania mine. In these experiments, the capability of the smart garment system to operate in a real environment was proved. Various experiments, including the validation of the localization, communication, and biometric functions of the smart garment, were conducted. Figure 18 and Figure 19 show the process of the installation of equipment before starting the real experiments in the Marini-Marmi mine in Italy.

Similar experiments regarding the sensors, the localization, and the communication components of the smart garment were conducted in the Kemi mine in Finland. The experiments took place in the warehouse area. This area was at a depth of 350 m from the surface. In this area, maintenance work on the machines is performed.

### 4.1. Validation Tests

#### 4.1.1. Air Quality Measurements (Smart Garment Hub)

The MiCS-6814 sensor is an analog device designed to detect three different gases: carbon monoxide (CO), nitrogen dioxide (NO_2_), and ammonia (NH3). It incorporates specific sensors for each gas. To capture the gas concentrations accurately, an analog-to-digital converter (ADC) is employed to measure the output voltage of the sensor. This voltage is then converted into sensing element resistance. The sensor’s manufacturer typically provides a logarithmic diagram that aids in converting the resistance values to precise gas concentrations [40].

The initial task involved deriving a mathematical expression linking the sensor’s output resistance (*Rs*) to the voltage, marked as Type 1 in the documentation. Following this, the next step was to compute the ratio of *Rs* to the sensing resistance in clean air (*R*0), denoted as Type 2. Subsequently, the third step involved calculating the gas concentration in parts per million (ppm), designated as Type 3.
$Rs\;=\;\frac{Vadc\;\cdot \;RL}{Vcc\;-\;Vadc}$$d\;=\;\frac{Rs}{R0}$$C\;=\;10^{\frac{\log d\;-\;b}{a}}$

The graphical representations within the diagrams vary depending on the gas concentrations. Therefore, it was necessary to determine the parameters ‘*a*’ and ‘*b*’, as well as various values found in Table 2, to accurately interpret the diagrams.

The subsequent step involved validating the measurement process. Initially, outdoor experiments were conducted in a clean-air environment. Additionally, publicly available climatological data [41] were utilized to supplement these tests.

In order to calibrate the smart garment gas sensors, measurements were gathered from the Kemi mine underground warehouse. The smart garment vest device was placed near Norlab’s P1 gas sensors in order to gather and compare the measurements for a period of two weeks. 

Kemi Mine was the ideal place to measure gases like NH_3_, NO_2_, and CO due to explosions and the heavy vehicles that move in underground passageways.

The smart garment has the ability to measure gases with the shown concentrations (Table 3). If the concentration of the gases is below or above these values, the garment returns the higher or the lower value.

**Figure 20:**
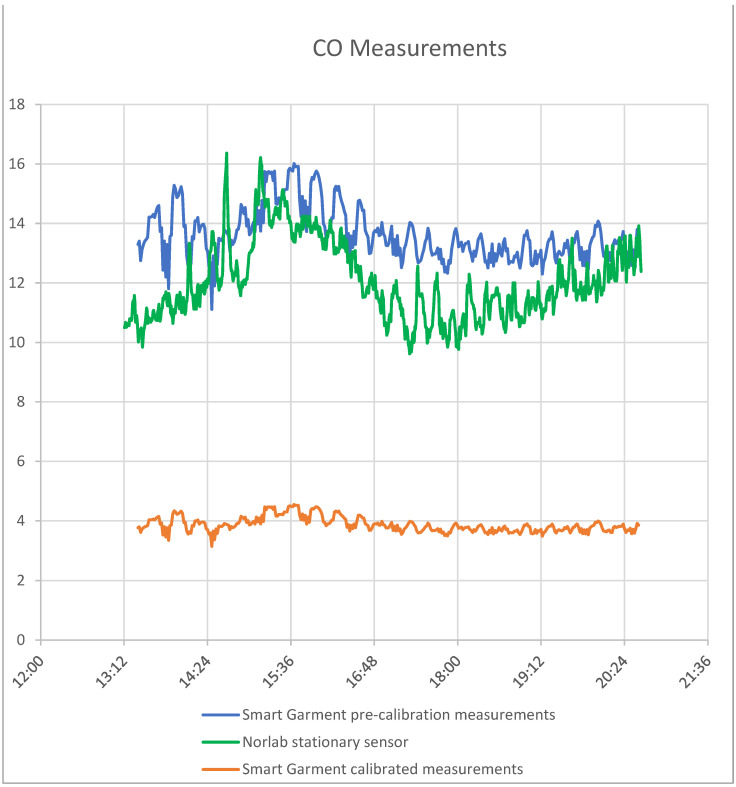
CO measurements (smart garment measurements, Norlab stationary sensor measurements, and calibrated measurements).

**Figure 21:**
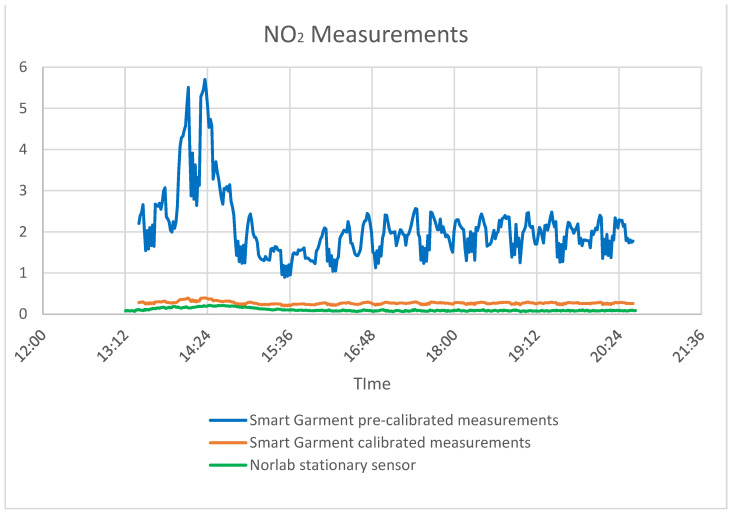
NO_2_ measurements (smart garment measurements, Norlab stationary sensor measurements, and calibrated measurements).

**Figure 22:**
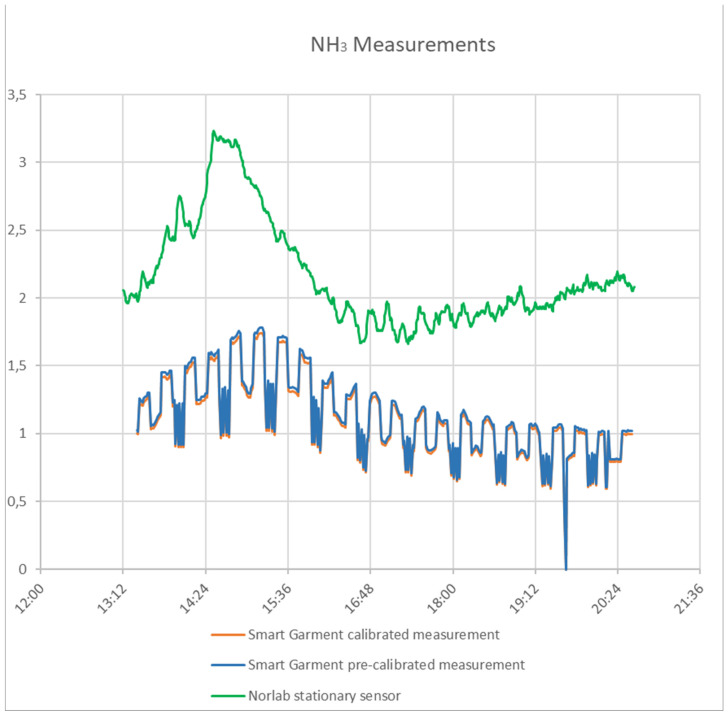
NH_3_ measurements (smart garment measurements, Norlab stationary sensor measurements, and calibrated measurements).

The sensor used to measure gases utilizes the change in resistance when a specific gas is present. Resistance is affected by temperature and humidity. By calculating certain variables that depend on the sensor’s usage space, the sensor can be software calibrated. These variables were calculated in the specific mine with the help of Norlab data. Before the calibration with the P1 gas sensor, the smart garment device had a big difference in NO_2_ measurement in comparison with the P1 sensor. After the calibration, the accuracy was significantly improved. Figure 20, Figure 21 and Figure 22 show the environmental sensors’ accuracy and sensitivity before and after calibration. 

#### 4.1.2. Localization Capabilities Testing

The smart garment vest integrates an ultra-wideband (UWB) component alongside its sensors, forming the foundation of its localization system. Within this system, tags and anchors collaborate to precisely determine the wearer’s position within the environment. Tags, which are nodes attached to the smart garment, establish wireless communication with anchors, stationary reference points dispersed throughout the area. Using UWB signals, Tags measure the time it takes for signals to travel to and from anchors, employing Decawave’s Two-Way Ranging (TWR) method, a type of time-of-flight (ToF) technique. This time delay allows for accurate calculation of the distance between each tag and anchor. By communicating with a minimum of four anchors, tags effectively triangulate their position within the environment. The individual distance measurements from each tag to multiple anchors are then gathered and processed by a location engine. This engine meticulously analyzes the data, culminating in the precise determination of the tag’s location relative to the anchors. Given the pivotal role of these distance measurements in accurately pinpointing the wearer’s position, evaluating the performance of the UWB component is paramount. Both accuracy and range must be thoroughly assessed to ensure the reliability and precision of the localization solution. Such evaluation is indispensable for achieving optimal system performance and functionality.

The DW1000 IC is capable of transmitting using three different data rates (110 kbps, 850 kbps, 6.8 Mbps), which offer a trade-off between airtime minimization and receiver sensitivity, enabling the system designer to target high tag density deployments and power consumption minimization or increased communication distance. Furthermore, there are six RF channels from 3.5 GHz to 6.5 GHz supported. The selection of channel 1 (3.5 GHz) and 110 kbps data rate should, in theory, offer the longest range, while the combination of channel 5 and 6.8 Mbps is appropriate for high-density deployments and tag miniaturization. 

As a result, the following configurations have been tested:

- 110 kbps data rate at 3.5 GHz (channel 1);
- 6.8 Mbps data rate at 6.5 GHz (channel 5).

Having the above in mind, a custom firmware was developed in order to be able to tweak the IC parameters and gain access to the channel impulse response (CIR) register to visualize the impulse response seen by the receiver. The channel impulse response retrieved from the module allows a quantitative evaluation of the multipath clutter and, consequently, confidence in the reported range solution.

*Outdoor testing:* A pair of transceivers was initially deployed in an outdoor space, free of obstacles, to get a view of the channel response, which will serve as a reference of an ideal channel and, at the same time, evaluate the communication range of the devices. There is hardly any multipath to be noted, and the dominant pulse is not preceded by any clutter, a strong indication of a successful detection of the first pulse and therefore a correct measurement.

**Figure 23:**
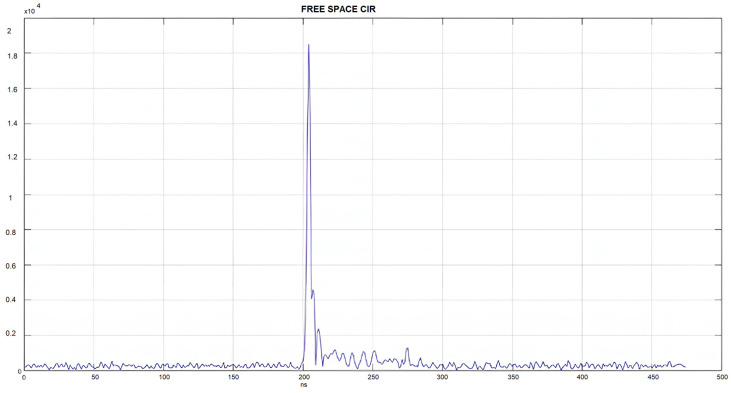
Outdoor channel impulse response.

The maximum communication distance between two nodes for the two different configurations mentioned earlier was then measured. According to the DW1000 IC’s dataset, a data rate of 6.8 Mbps results in a receiver sensitivity of −94 dBm at a 10% packet error rate, and a data rate of 110 kbps enables packet reception at −106 dBm. The combination of 6.5 GHz and 6.8 Mbps achieves a maximum communication range of around 30 m, while when channel 1 was selected (3.5 GHz) along with a 110 kbps data rate, we were able to successfully receive packets at distances in excess of 100 m, as indicated in Figure 23. It should be noted that the range limitation was a result of the transmitter and receiver height above ground in accordance with the two-ray model, which is applicable here.

**Figure 24:**
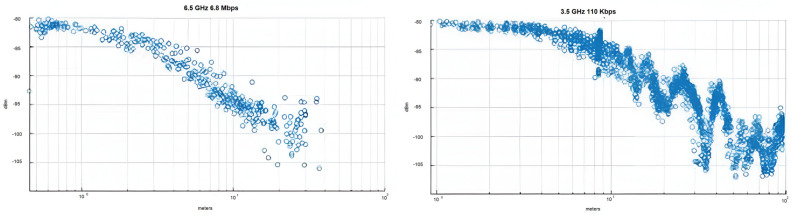
Received signal strength versus distance for various transceiver configurations.

The maximum communication distance between two nodes for the two different configurations was then measured. According to the DW1000 IC’s dataset, a data rate of 6.8 Mbps results in a receiver sensitivity of −94 dBm at a 10% packet error rate, and a data rate of 110 kbps enables packet reception at −106 dBm. The combination of 6.5 GHz and 6.8 Mbps achieves a maximum communication range of around 30 m, while when channel 1 was selected (3.5 GHz) along with a 110 kbps data rate, we were able to successfully receive packets at distances in excess of 100 m, as indicated in Figure 24. It should be noted that the range limitation was a result of the transmitter and receiver height above ground in accordance with the two-ray model, which is applicable here.

*Indoor Line-of-Sight*: For the indoor measurements, the office hallway was selected to perform measurements in an environment with severe multipath, a phenomenon similar to what is expected to be encountered inside a mine or in the machine. The gray doors are made of metal, as are most of the structures other than the windows and the walls. The narrow shape of the hallway makes the pulses emitted by the UWB transmitter meet the walls at shallow angles, causing grazing incidence, which results in most of the energy being reflected. The two UWB modules were installed on top of the tripods.

**Figure 25:**
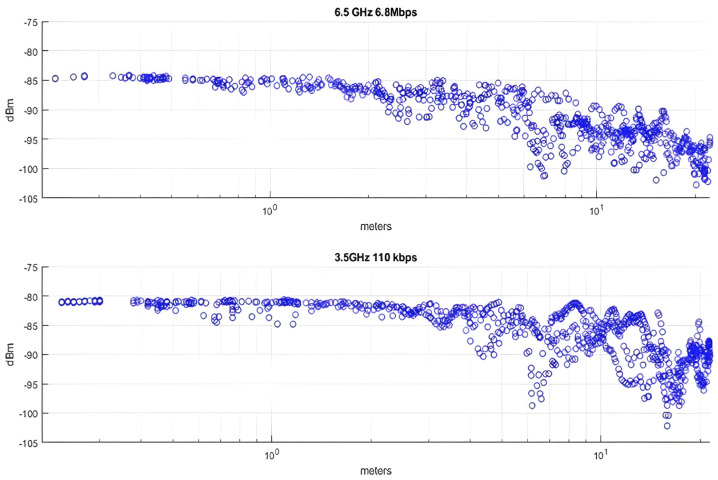
Received signal strength for indoor line-of-sight localization.

The received signal strength versus distance for the two configurations is presented in Figure 25. Both configurations offer adequate coverage of the 20-m-long hallway, with the low-data-rate configuration performing better.

**Figure 26:**
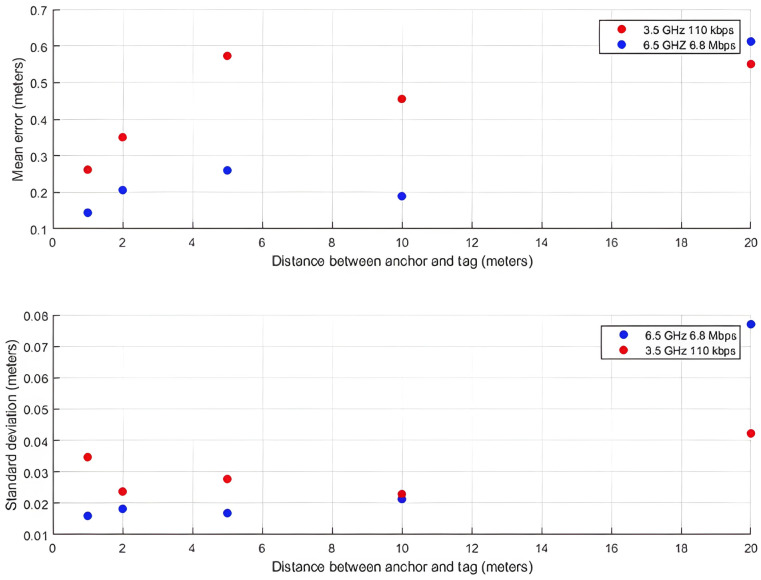
Distance measurements’ mean error and standard deviation values.

Next, the two modules were placed at predefined spots, separated by 1, 2, 5, 10, and 20 m, and the distance readings were compared against the ground truth. Figure 26 presents the mean error and standard deviation of these measurements. The configuration at 6.5 GHz frequency and 6.8 Mbps data rate appears to give better performance.

At the 20-m mark, where the mean error reached 60 cm, the receiver was near a metal door (45 cm away) at the end of the hallway. While the 3.5 GHz and 110 kbps configurations display comparable error values at other points as well, for the 6.5 GHz and 6.8 Mbps configuration, this error value is double any other error recorded. To further investigate this case, we took a look at the channel impulse response at 2 and 20 m, which can be seen in the figure below, where in both cases the multipath components were more powerful than the direct pulse due to the metal door at end of the hallway, which acts as a reflector directing the RF energy towards the receiver. The main difference between the measurements at 2 and 20 m is that the reflection following the first pulse at 20 m has double the energy of the one at 2 m, which leads the edge detection microcode running onboard the DW1000 to set a higher pulse detection threshold, which causes the error. Yet, a mean error of 60 cm is manageable and not a source of concern.

**Figure 27:**
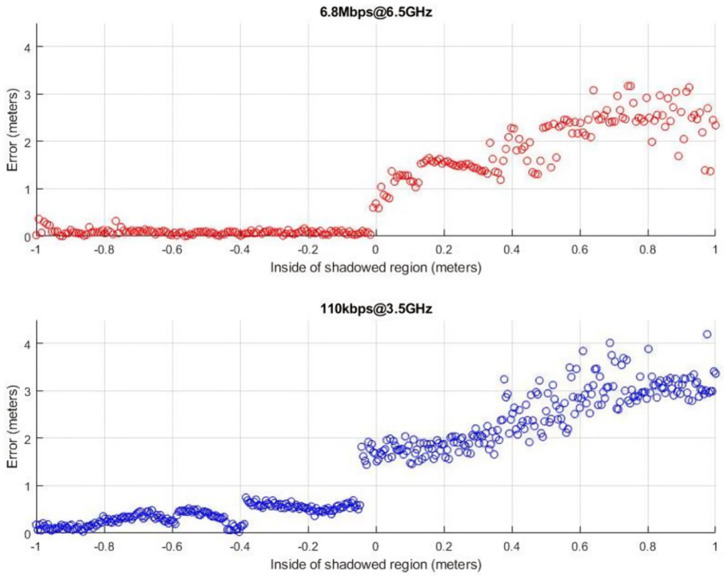
Distance measurements for non-line of sight conditions at a 15-m true distance.

*Indoor Non-Line of Sight:* Finally, we took a look at the impact of non-line-of-sight conditions on the solution’s localization accuracy. To that end, we placed the anchor and tag 15 m apart and then gradually moved the tag behind a corner while keeping a fixed distance between the two nodes and logging the results. Initially the distance measurements were near the real distance, but as the tag was moving deeper into the shadowed region ever longer distances were reported. This is due to the tag’s failure to receive the pulse corresponding to the blocked LoS path and using instead a multipath component to calculate the distance. The more reflections the pulse had undergone, the greater the distance overestimation. Both configurations show errors in the range of meters, as depicted in Figure 27.

**Figure 28:**
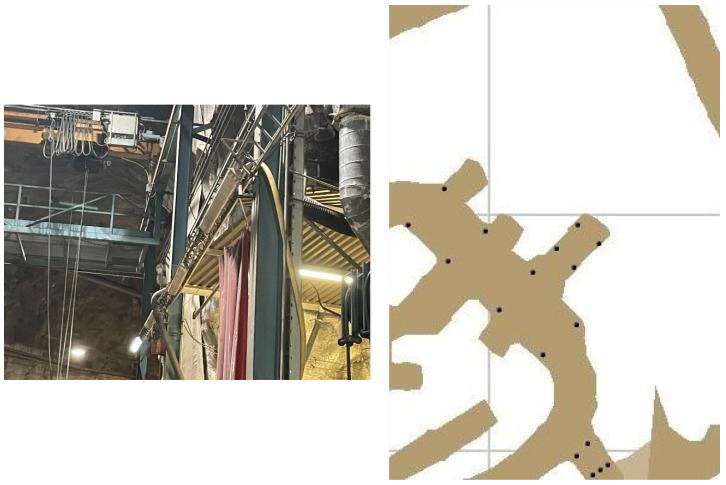
Kemi mine—anchor installation and localization map.

*Kemi mine testing:* During the whole shift, the miners are working in this area, so it is easy to track their location. In order to place the anchors, we performed some measurements of the dimensions of the area. The anchors were placed on the metal columns by using strong magnets in order to avoid the drilling of the walls, which can affect the stability of the galleries (Figure 28). In these areas, there were two places that challenged the localization procedure. In the area with the number one, there are big vehicles that interfere with the signal’s ability to propagate properly. In order to confront this problem, the anchors were installed at a height of 4 m on the metal columns of the crane bridge. The second area was material storage; therefore, it had metal shelves along the aisles, and there was also a mezzanine. So, it was necessary to complete the most accurate localization by using the fewer anchors’ devices. In order to solve this problem, four anchors were installed on the front at a height of 2.7 m and two on the ground floor at a height of 2.3 m in order to be near the floor of the loft. With this topology, accurate localization was achieved. In the third area, there was no metal column; the only place that the anchors could be installed was the metal containers. Thus, two anchors were installed in front of them at a height of 2.6 m, and another two inside every container. With these topologies, the whole area between the container and the anchor with the numbers 1 and 5 was successfully covered. The complete grid is presented in Figure 28.

*Titania testing:* Similarly, a testing campaign took place in the Titania mine. The localization system test was conducted in the garage of the mine, where the workers are repairing heavy machinery like haul trucks and wheel loaders. The testing ground was chosen due to the fact that there are many workers there during the day.

**Figure 29:**
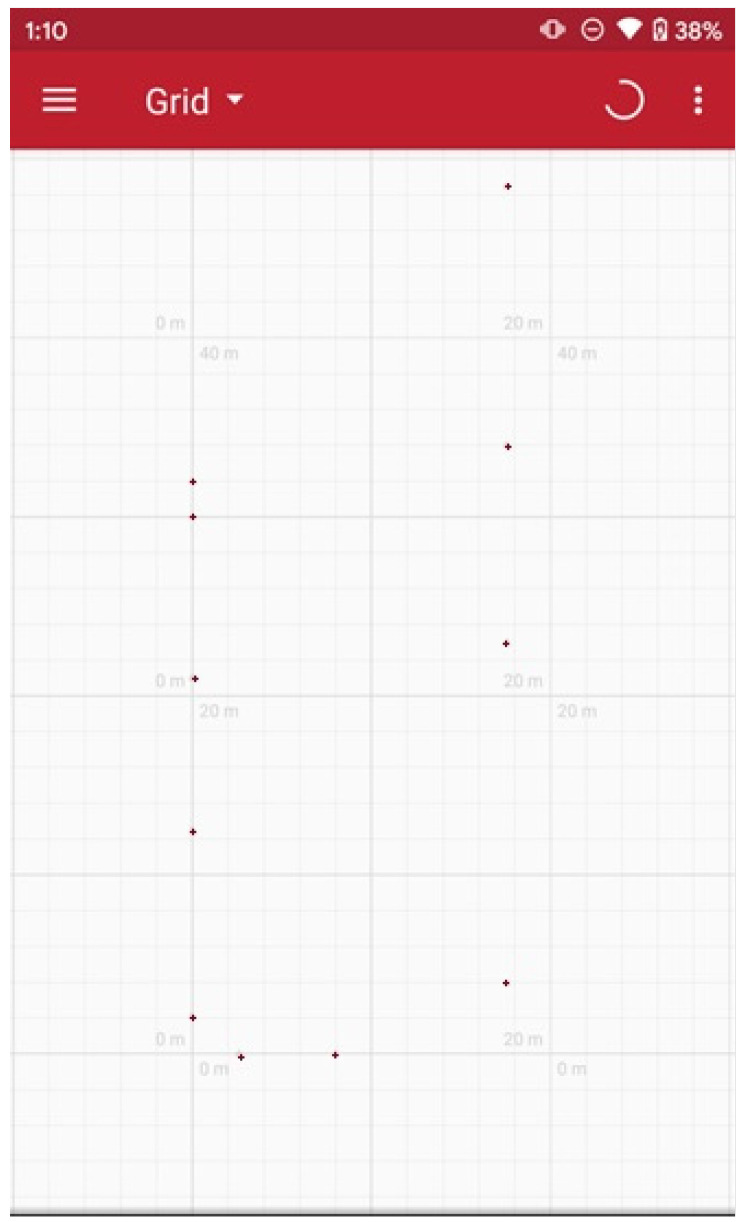
Titania mine—anchor grid according to the localization application.

The localization test was held in two different places that were separated by a big metal door. The dimensions of the whole are 17.56 m in width, length of 80.5 m, and height of 15 m. At first, the UWB anchors were placed at a distance of 15 m from each other and at a height of 2.46 m. The distances and the fact that there was a lot of metal had a negative effect on the measurement procedure. We were therefore forced to place the anchors at smaller distances in order for the garment to be able to communicate with at least three anchors. The second set of tests took place in the second room, which is 32 m in length. From the second test was decided that the anchors should be placed at a higher position from the ground and should be closer to each other. Table 4 summarizes the measurements from the localization experiment, while Figure 29 shows the grid of anchors.

#### 4.1.3. Noise Level Testing

**Figure 30:**
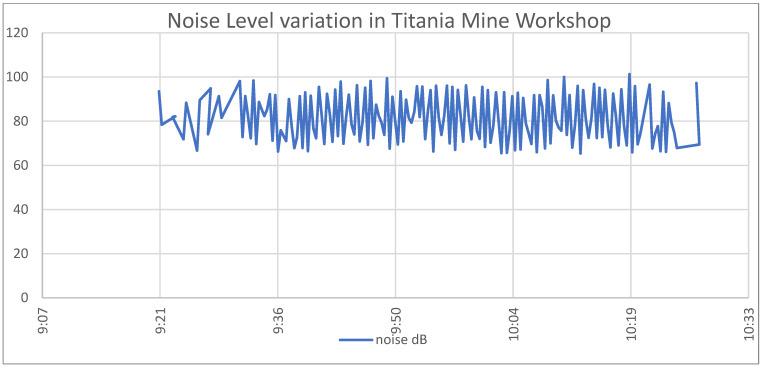
Smart garment noise level measurements.

During the experiments in the Titania mine, all the capabilities of the garment were enabled, and data were gathered from every individual sensor. This place was suitable for testing the environmental sensors due to the high concentration of the examining gases and the noise that the machinery was producing. The noise level fluctuated around 80 dB. These measurements have been validated by using the Decibel X (version 9.7.0), a software that uses the mobile phone microphone. The fluctuation of the noise level is presented in Figure 30.

#### 4.1.4. Health Status Monitoring Experiments

**Figure 31:**
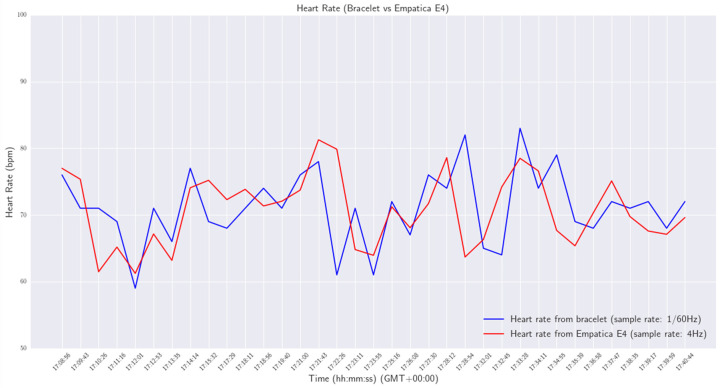
Heart rate—wristband versus Empatica E4.

**Figure 32:**
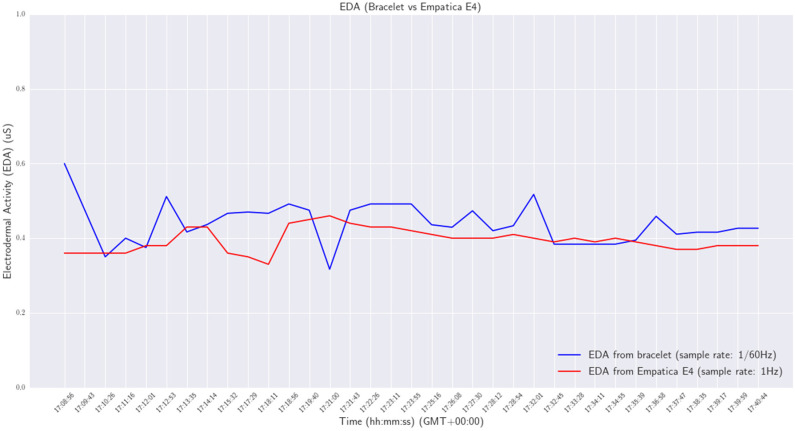
EDA—wristband versus Empatica E4.

**Figure 33:**
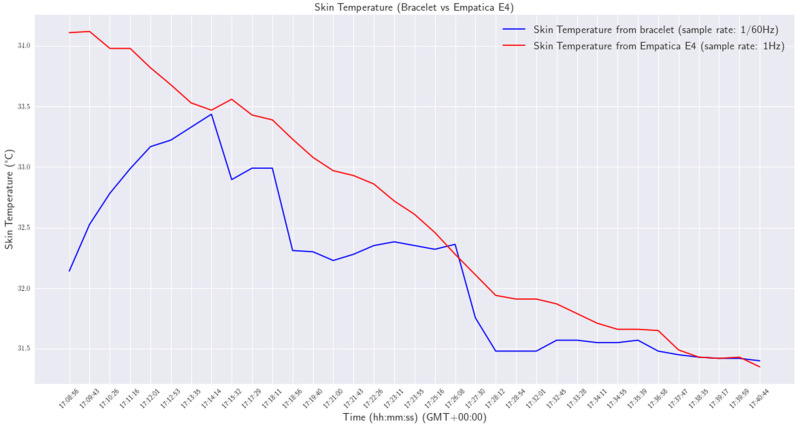
Skin temperature—wristband versus Empatica E4.

Various experiments were conducted in order to validate the functionality and reliability of the smart wristband. All experiments were performed in a laboratory environment. Initially, the wristband was tested as a standalone device to ensure that the developed firmware was working properly and to assess the reliability of acquiring measurements from the various personal health status sensors. Afterward, the smart wristband performance was compared with a commercial Class 2A medical wristband (Empatica E4 [42]). Specifically, the heart rate, EDA, and skin temperature of a person were measured at the same time by the smart wristband on the right wrist and the E4 wristband on the left. The experiment lasts for approximately 32 min. The sample rate of the wristband was configured at one measurement per 60 s (0.17 Hz), while the sample rate of the E4 was at default 4 Hz for heart rate and 1 Hz for the other vital signs. The results are shown in Figure 31, Figure 32 and Figure 33.

From the results, a mean difference of 4.52 bpm between the heart rates provided by the two devices was obtained.

From the results, a mean difference of 0.084 μS between the skin conductance measurements provided by the two devices was obtained.

From the results, a mean difference of 0.48 between the skin temperature measurements provided by the two devices was obtained.

**Figure 34:**
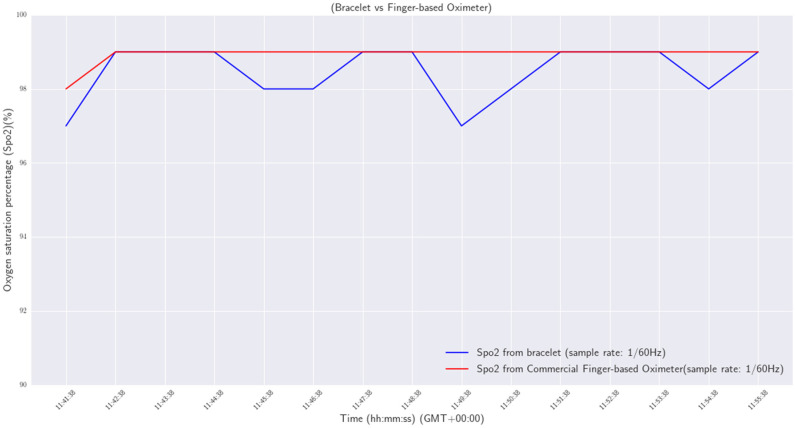
SpO_2_—wristband versus commercial finger-based oximeter.

For the SpO_2_ measurements, a commercial finger-based oximeter was employed, and the oxygen saturation percentages provided by the oximeter and the smart wristband were compared. Specifically, a volunteer simultaneously wore the wristband on the left wrist and the commercial oximeter in his index finger. Then, he stayed still for 15 min to measure his oxygen saturation level. The sample rate for both devices was one measurement per minute (1/60 Hz). The results are shown in Figure 34.

From the results, a mean difference of 0.467 between the SpO_2_ measurements provided by the two devices was obtained.

#### 4.1.5. Communication Capabilities Testing

*Bluetooth Low Energy (BLE):* In the second phase, communication with other subsystems and specifically with the smart garment hub was tested. During these tests, the wristband managed to successfully establish a BLE connection with the smart garment hub in the maximum range of ~3 m and transmitted all the acquired data via multiple BLE packets (15 packets of 100 raw PPG data, 1 packet including all the biometrics) and custom services, respecting the BLE’s GATT standard. After the smart garment received all the data successfully, it published them in JSON format to the MQTT broker and the IIoT platform.

**Figure 35:**
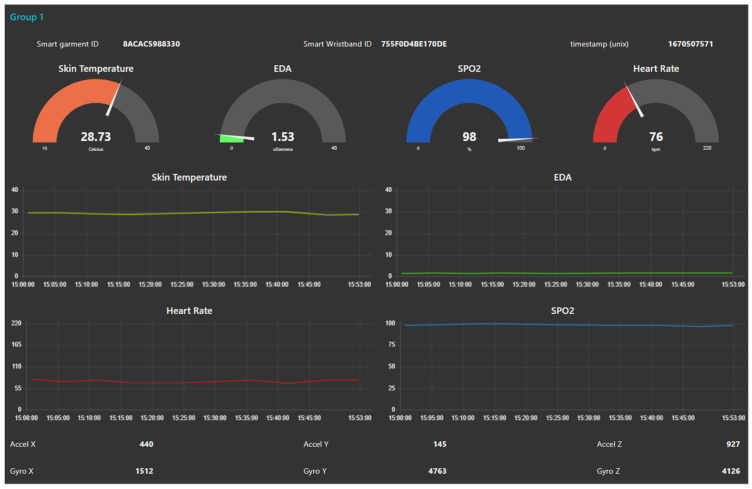
Dashboard visualizing biometric data acquired from the wristband.

Within the context of the last performed tests, multiple workers agreed to wear a wristband on the wrist and a garment hub on their belt for a whole working day (~8 h). Consequently, their biometric data, which include all the measurements acquired by the aforementioned sensors, were gathered by the smart garment hub. Then, the data were transmitted to the IIoT platform via the MQTT protocol, where they were stored permanently in the database. Biometric sample data acquired from the wristband during these tests and stored in the database of the IIoT platform are presented on a dashboard in Figure 35. Furthermore, wristband devices were distributed in real fields in mines, and the biometric data from the mine workers were continuously transmitted to the IIoT platform. 

*Wi-Fi:* In order to transmit the data from the vest to any communication infrastructure, the Wi-Fi protocol is used. The ESP32 MCU has an integrated Wi-Fi module that works at 2.4 GHz frequency. By using different access point devices with predefined Wi-Fi SSIDs and passwords, we were able to connect to the communication infrastructure and the Internet. Various experiments were conducted to confirm the successful communication, using the MQTT publish–subscribe protocol, between the field and the IIoT platform over Wi-Fi.

#### 4.1.6. Speech Recognition Experiments

A PDM microphone is used for collecting the samples for a period of 5 s. In order to avoid oversampling and to reduce the power consumption of the device, a press button is used by the user so the device starts sampling before the user pronounces a message. When the sampling starts, the device informs the user that it is working with a characteristic sound. After that, the device detects the word pronounced by the user, utilizing an ML model, and sends a message via BLE to the vest device via a specific service. The ML model created is implemented in the NRF52840 CPU for the Zephyr RTOS [43]. The Edge Impulse automatically extracts the model for the microcontroller in C++ programming language. The earplug device acts as a BLE service, and the smart garment vest device is a BLE client. Each word detected by the model is characterized by a specific number. The vest receives the command and sends the message to the IIoT Platform via the MQTT protocol.

The neural network was trained using the Google Speech Commands dataset, which includes 105,000 sound samples for 30 different words. These samples were recorded at a frequency of 16 kHz using a pulse density modulation (PDM) microphone. The microphone in the device also operates on PDM technology, converting sound signals into 1-bit digital samples, where the density of pulses represents the amplitude of the analog signal. The machine learning model was created using the Edge Impulse method, a cloud-based platform that simplifies model training. Before uploading the dataset to Edge Impulse, noise was added to simulate real-world conditions.

For feature extraction, the mel-frequency cepstrum (MFC) method was chosen. This technique represents the short-term power spectrum of sound by transforming a log power spectrum onto a nonlinear mel scale of frequency. The resulting vector captures temporal changes to reflect the dynamic nature of speech. The MFCC algorithm involves five steps: spectrum analysis using the fast Fourier transform, calculation of the mel frequency spectrum through filtering with a filter bank, taking the logarithm of intensity values, computing the Cepstral coefficients via Discrete Cosine Transform, and expanding the vector with first- and second-order derivatives to capture temporal changes. After applying the MFCC algorithm, neural network training was conducted using Keras, an open-source Python library designed for fast experimentation with deep neural networks. Keras prioritizes user-friendliness, modularity, and extensibility, making it suitable for developing and training complex models efficiently.

In order to check if the platform works, experiments were performed using two serial port monitors for the earplugs and the vest device to check if the first detects the word and then if the message is transmitted to the second. As presented in Figure 17, from the serial port monitor of the earplugs, it detects the word “zero” and transmits the message via the BLE. Afterward, the vest device receives the message and transmits it via MQTT to the IIoT platform. Instead of sending audio over the BLE interface, a number of commands are sent to the smart earplugs, and they are locally translated to audio.

In order to inform the users about various situations during their work hours, the earplugs have a speaker. In order to maintain consumption at low levels, the information which is transmitted between the vest and the earplugs has been limited to a small number of predefined messages. Each message refers to a specific audio track. Each device is equipped with an SD card on which audio tracks are stored in a .wav audio file format. The reason that this format was chosen is that it does not need any conversion, and it can be directly reproduced using the I^2^S protocol and a sound module.

The I^2^S protocol is a serial bus interface specifically designed for communicating digital audio data between integrated circuits. It sends audio data using a pulse code modulation. The wiring protocol has at least three lines: the bit clock, the word select, and the data line. Using the word select, a bus can specify in which audio channel the data will be transferred. In these implementations, a stereo audio channel was used.

The architecture of the alert notification mechanism for the smart garment is displayed in Figure 17. The alert is transmitted via a Kafka broker. The notification from the Kafka broker is transmitted via the MQTT protocol to the smart garment vest access point device. This device transmits the alert message via the BLE protocol to the earplugs device in order to play the predefined sound file and notify the end user. Through a series of tests, we have confirmed that the earplugs, the vest, and the IIoT platform are working as expected, and an alert can be transmitted to the end user.

#### 4.1.7. Alert System Validation

In addition to evaluating the technical components of the system, a series of tests were conducted to validate the reliability of the alert decision-making system. These tests involved planning and executing various emergency scenarios in real mines, focusing on functionality, accuracy verification, and worker acceptance.

Prior to initiating the validation procedure, workers underwent training to familiarize themselves with the smart garment equipment and understand the responses of the alert system. This training was crucial to ensure that workers could interpret alert signals correctly.

The validation process began by confirming that all system functionalities operated as intended. Multiple emergency scenarios, primarily focusing on gas leaks and harmful air conditions, were simulated to trigger corresponding alerts. The accuracy of these alerts was validated by comparing them against predefined criteria for emergency situations, ensuring alerts were issued only when necessary. The earplugs served as the core device for delivering alerts, so their effectiveness in transmitting alert sounds to workers, even in noisy environments, was thoroughly tested during these experiments.

Feedback was collected from workers through personal interviews, surveys, and questionnaires to assess their overall experience with both the equipment and the alert system’s performance.

Based on the gathered feedback and ongoing monitoring of real mine conditions, the alert system undergoes continuous improvement. Future efforts will aim to automate the alert process by employing machine learning models to analyze data from various sensors installed across different mine locations. This automation would enable the system to issue appropriate alerts without human intervention, further enhancing safety measures in mining environments.

## 5. Discussion

In our study, we placed a strong emphasis on integrating user feedback and conducting an in-depth user study to thoroughly assess the practicality and effectiveness of the proposed integrated system for monitoring worker health and safety in mining environments. We actively engaged with stakeholders, including miners, safety officers, and mine management, to gain valuable insights into their specific needs, preferences, and concerns. Through collaborative discussions and iterative feedback sessions, we gained a deeper understanding of the challenges faced in mining operations and tailored the system design to address these real-world complexities.

Additionally, we conducted a detailed user study to empirically evaluate how well the system performed in actual mining environments. This involved deploying the system on-site and collecting feedback through various methods such as surveys, interviews, and direct observations. By actively involving end users in the evaluation process, we were able to assess the system’s ease of use, reliability, and impact on safety practices. The findings from our user study guided us in refining and improving the system. By carefully analyzing user feedback and observations, we identified areas where enhancements were needed to better meet the needs of mining industry workers.

Our focus on user-centered design not only enhances the credibility of our research but also increases the likelihood of successful adoption and implementation of the system in mining operations. By prioritizing the perspectives and insights of end users, we have developed a solution that is closely aligned with the practical realities and safety requirements of the mining industry. Thus, the importance of incorporating user feedback and conducting thorough user studies in the development of technology solutions for complex industrial environments like mining has been shown. By actively involving stakeholders throughout the process, we have created a system that not only meets technical requirements but also addresses the day-to-day challenges faced by workers in the mining sector.

## 6. Conclusions

In this paper, an end-to-end IoT system for supporting workers in harsh environments such as in mining industries is presented. The full IoT solution includes both edge devices worn by the workers in the field and a remote cloud IoT platform, which is responsible for storing and efficiently sharing the gathered data in accordance with regulations, ethics, and GDPR rules. Extended experiments conducted to validate the IoT components both in the laboratory and in the field proved the effectiveness of the proposed solution in monitoring the real-time status of the workers in mines.

Future work will include the scaling of the proposed approach by using a larger number of devices in each mine and the adaptation of the data rates in a way that will minimize the data stored in the cloud while retaining the high performance of the end-to-end solution. The collaboration of the mines in this process with the simultaneous willingness of the workers to wear the equipment will be the key to the success of the aforementioned approach.

## Figures and Tables

**Table 1:** Edge processing function running on each device.

*Wristband*	*Vest*	*Ear-Plugs*
Heart rate estimation [17,18,19,20,21,22]	Localization [23,24,25,26,27,28]	Voice recognition—ML Model [29,30,31]
Oxygen saturation computation [32,33]	Air quality measurements	Alert commands
Skin temperature		
Movement detection		
Galvanic skin response		

**Table 2:** Determination of a and b parameters.

CO (ppm)	*a*	*b*
1–10 ppm	−0.8451	0.544
10–100 ppm	−0.8539	0.5229
100–1000 ppm	−0.8451	0.5353
**NO_2_ (ppm)**	a	b
0.001–0.1 ppm	0.9652	0.7434
0.1–1 ppm	1.0348	0.8129
1–10 ppm	0.3785	0.8129
**NH_3_ (ppm)**	a	b
1–10 ppm	−0.5414	−0.097
10–100 ppm	−0.5292	−0.1091
100–1000 ppm	−0.5708	−0.0259

**Table 3:** Smart garment gas sensors’ range.

CO	1–1000 ppm
NH_3_	1–165 ppm
NO_2_	1–6.5 ppm

**Table 4:** Measurements (in cm) from the localization experiment.

X	Y	Z
3471	2094	−1027
4996	2761	−504
3999	6859	244
4024	7025	1010
3887	8453	2490
4534	6599	−398
4521	7043	1709
6205	4212	−112
6078	8820	−1034
5757	9032	5296
6459	8502	1232
6573	8658	379
7092	13708	1085
6875	13879	1349

## Data Availability

Data are contained within the article.
